# Ultrasound-Activated Microbubbles for Precision Chemo-Radioenhancement in Prostate Cancer: Sonoporation, Therapeutic Synergy, and Translational Pathways

**DOI:** 10.3390/cancers18183067

**Published:** 2026-09-21

**Authors:** Firas Almasri, Raffi Karshafian

**Affiliations:** 1 College of Engineering and Sciences, International University of Science and Technology in Kuwait, Ardiya 92400, Kuwait; 2 Center for Education Studies, University of Warwick, Coventry CV4 7AL, UK; 3 Department of Physics, Toronto Metropolitan University, Toronto, ON M5B 2K3, Canada; karshafian@torontomu.ca; 4 Institute for Biomedical Engineering, Science and Technology (iBEST), a Partnership Between Toronto Metropolitan University and St. Michael’s Hospital, Toronto, ON M5B 1T8, Canada

**Keywords:** prostate cancer, ultrasound-stimulated microbubbles, sonoporation, docetaxel, radiosensitisation, chemo-radiotherapy, cavitation, drug delivery, tumor vasculature, translational oncology

## Abstract

**Background/Objectives:** Radiotherapy and docetaxel are mainstays of prostate cancer management, yet both are constrained-radiotherapy by normal-tissue tolerance and docetaxel by systemic toxicity and acquired resistance. Ultrasound-activated microbubbles (USMB) convert acoustic energy into spatially localized mechanical bioeffects that can potentiate both. This review examines USMB as a precision chemo- and radio-enhancer in prostate cancer. **Methods:** We conducted a scoping review with mechanistic and translational synthesis, informed by PRISMA-ScR reporting principles. We identified sources through a structured bibliographic search and supported it with source-level verification. Heterogeneity in model systems, acoustic parameters, treatment regimens, and outcomes precluded meaningful quantitative pooling; we therefore synthesized the evidence narratively. **Results:** Two distinct mechanisms recur: sonoporation-cavitation-driven permeabilization that increases intracellular docetaxel delivery and acid sphingomyelinase (ASMase)-mediated ceramide-driven endothelial perturbation that sensitizes tumor vasculature to radiation. In prostate models, the three modalities enhance one another, and the triple combination produces the largest reported increases in clonogenic death, apoptosis, and regression in selected immunodeficient PC3 models. These effects were achieved at reduced experimental drug and radiation doses, a potential dose-sparing benefit that remains a preclinical hypothesis. Human data, although still outside the prostate, provide early support for feasibility and acceptable safety in selected USMB-enhanced settings. **Conclusions:** The rationale is coherent and the preclinical prostate signal consistent, but the evidence remains predominantly preclinical, PC3-dominated, and heterogeneous. USMB is best understood not as a drug-delivery technique but as a tunable sensitization platform whose outcome depends on acoustic dose, vascular state, and sequencing; parameter standardization, prostate-specific delivery, and cautious early-phase trials remain prerequisites for translation.

## Simple Summary

Prostate cancer is treated with radiotherapy and, in advanced disease, with the chemotherapy drug docetaxel. Both work, but radiation dose is limited by damage to nearby healthy tissue, and docetaxel reaches the whole body, causing side effects as tumors often become resistant. This review examines a physical strategy that may increase the local effectiveness of both treatments without increasing systemic drug exposure or the radiation dose to healthy tissue. Tiny gas bubbles, already used as ultrasound contrast agents, are injected into the bloodstream and made to oscillate and collapse by focused ultrasound directed at a defined prostate treatment volume. This transiently opens cell membranes and briefly disrupts tumor blood vessels, increasing drug uptake and amplifying radiation effects, with the intention of confining these effects to the insonated volume. We synthesize laboratory and animal evidence and outline what must be resolved before testing in patients.

## 1. Introduction

Prostate cancer remains one of the most commonly diagnosed malignancies in men, and the clinical challenge it poses is less often one of initial control than of doing so without exacting a lasting price in urinary, bowel, and sexual function, and without eventually meeting treatment resistance [1,2]. For localized and locally advanced disease, external beam radiotherapy, frequently combined with androgen deprivation therapy, is a standard curative-intent option whose oncological results rival surgery and is recommended as such in contemporary international guidelines [1,3]. One central limitation is both physical and biological: the dose that will reliably sterilize the tumor is bounded by the tolerance of the immediately adjacent rectum, bladder, and urethra. Dose escalation improves biochemical control but, as a Cochrane synthesis of nine randomized trials in more than five thousand men has shown, buys little or no improvement in prostate-cancer-specific or overall survival while increasing late gastrointestinal toxicity [4]. Compounding this ceiling is intrinsic and acquired radioresistance, now understood to arise from a web of DNA damage repair, hypoxic, and signalling programs that blunt the effect of a given physical dose [5,6], and the clinical reality that a substantial minority of men relapse locally and face difficult salvage decisions after primary radiotherapy [7,8]. Efforts to raise prostate radiosensitivity pharmacologically through DNA-repair inhibition, androgen-axis manipulation, and related targets are an active field in their own right [9,10]. That plateau is the practical motivation for a complementary, physical route to radiosensitization: a way to raise the biologically effective dose inside the tumor without raising it in surrounding tissues.

Systemic therapy faces the mirror image problem. Docetaxel, a taxane that stabilizes microtubules and blocks mitosis, was the first cytotoxic agent to prolong survival in metastatic castration-resistant prostate cancer [11,12], and its subsequent movement into the hormone-sensitive setting, established by the CHAARTED and STAMPEDE trials, made early docetaxel a component of standard care for many men with metastatic disease [13,14]. Nevertheless, its benefit is delivered systemically and unevenly: the response is limited by dose-dependent toxicity (neutropenia, neuropathy, fatigue) and, almost invariably in advanced disease, by the emergence of resistance [15,16]. The mechanisms are diverse and increasingly well mapped, spanning drug efflux through ABCB1/P-glycoprotein, tubulin and microtubule-regulator alterations, androgen-receptor signaling, and transcriptional and non-coding RNA programs, including NOTCH3 and miRNA networks [17,18,19,20]. Several of these converge on efflux-mediated exclusion of drugs from the cell, which is precisely the barrier that a method raising intracellular, not merely circulating, drug concentration is positioned to bypass. Against this backdrop, docetaxel remains a fixture of the treatment landscape even as newer agents accumulate around it, keeping the question of how to make each dose more clinically effective alive [21,22]. Improving the fraction of drug that actually reaches and enters tumor cells, and doing so preferentially within the prostate, would address both halves of that problem-toxicity and resistance at once.

Ultrasound-activated microbubbles sit precisely at this intersection. Throughout this review, the terms “ultrasound-activated” and “ultrasound-stimulated” microbubbles denote the same intervention-intravascular microbubbles driven into oscillation or collapse by an external therapeutic ultrasound field-for which the single abbreviation USMB is used; where cited studies employed other labels, such as ultrasound-targeted microbubble destruction (UTMD), the original term is retained only in the table entries reporting those studies. Microbubbles, micron-scale, gas-filled spheres stabilized by a lipid or protein shell, were developed and are licensed as intravascular ultrasound contrast agents, and their safety profile in diagnostic use is well established [23]. When exposed to a focused ultrasound field, they do more than scatter sound: they oscillate, and at sufficient acoustic pressure they collapse, converting acoustic energy into concentrated local mechanical forces-shear, microstreaming, and jetting-at the scale of individual cells and capillaries [24]. Because the bubbles are confined to the vasculature and the ultrasound can be steered to a defined volume, these bioeffects are expected to be spatially localized to the insonated region. Two consequences are therapeutically relevant. First, sonoporation transiently permeabilizes cell membranes and vessel walls, increasing the uptake of co-administered drugs into the insonated region [25]. Second, mechanical perturbation of the endothelium can trigger a ceramide-dependent apoptotic cascade that sensitizes tumor blood vessels to radiation [26]. In other words, the same agent offers a physical route to both chemo-enhancement and radio-enhancement, targeted to where the beam is placed.

This review examines that proposition specifically for prostate cancer, a disease unusually well suited to the approach: it is anatomically accessible to transrectal or transperineal ultrasound, it is routinely treated with both radiotherapy and docetaxel, and it has served as the principal model in which USMB radioenhancement has been developed. We first set out the therapeutic context that creates the need-the limits of radiotherapy and of docetaxel-and the rationale for local sensitization (Section 2). We then build the mechanistic argument from the physics of microbubble behavior and sonoporation (Section 3 and Section 4) through chemo-enhancement (Section 5), radio-enhancement (Section 6), and their combination in triple therapy (Section 7), before turning to the parameters that govern the effect (Section 8), the prostate-specific preclinical evidence (Section 9), and the imaging that could guide and monitor treatment (Section 10). Section 11 and Section 12 weigh the translational barriers and map a realistic path toward clinical evaluation. Throughout, our aim is synthesis and appraisal rather than enumeration: to distinguish what the evidence supports firmly from what remains mechanistically plausible but unproven, and to be explicit that most of what follows is preclinical and that the interest the field generates has to be matched against the modest size, methodological heterogeneity, and narrow provenance of much of the underlying data. The organizing argument, developed across the sections that follow, is that USMB chemo-radioenhancement is best understood not as a single drug delivery technique but as a tunable physical sensitization platform whose biological outcome depends on acoustic dose, vascular state, treatment sequence, and tumor context-a framing with direct consequences for how the approach must be optimized, monitored, and, eventually, tested in patients.

## 2. Materials and Methods

This review was conducted as a scoping review with mechanistic and translational synthesis, informed by PRISMA-ScR (Preferred Reporting Items for Systematic Reviews and Meta-Analyses extension for Scoping Reviews) reporting principles [27]. We deliberately chose a scoping design over a systematic review with meta-analysis: it is the appropriate methodology for mapping a heterogeneous, largely preclinical evidence base and characterizing mechanisms, parameter dependencies, and translational gaps, whereas quantitative pooling was judged inappropriate for the reasons set out in Section 2.4. The review was not conducted under a registered protocol. The research question, search strategy, and eligibility criteria were defined before screening began and are reported in full in Section 2.1 and Section 2.2, but the protocol was not lodged in a public registry, and the review therefore has no a priori registration record. The absence of prospective registration is acknowledged as a methodological limitation in Section 12, and prospective registration is recommended for future reviews in this area. The completed PRISMA-ScR checklist for this review is provided as Supplementary Table S1.

### 2.1. Search Strategy and Information Sources

**Figure 1:**
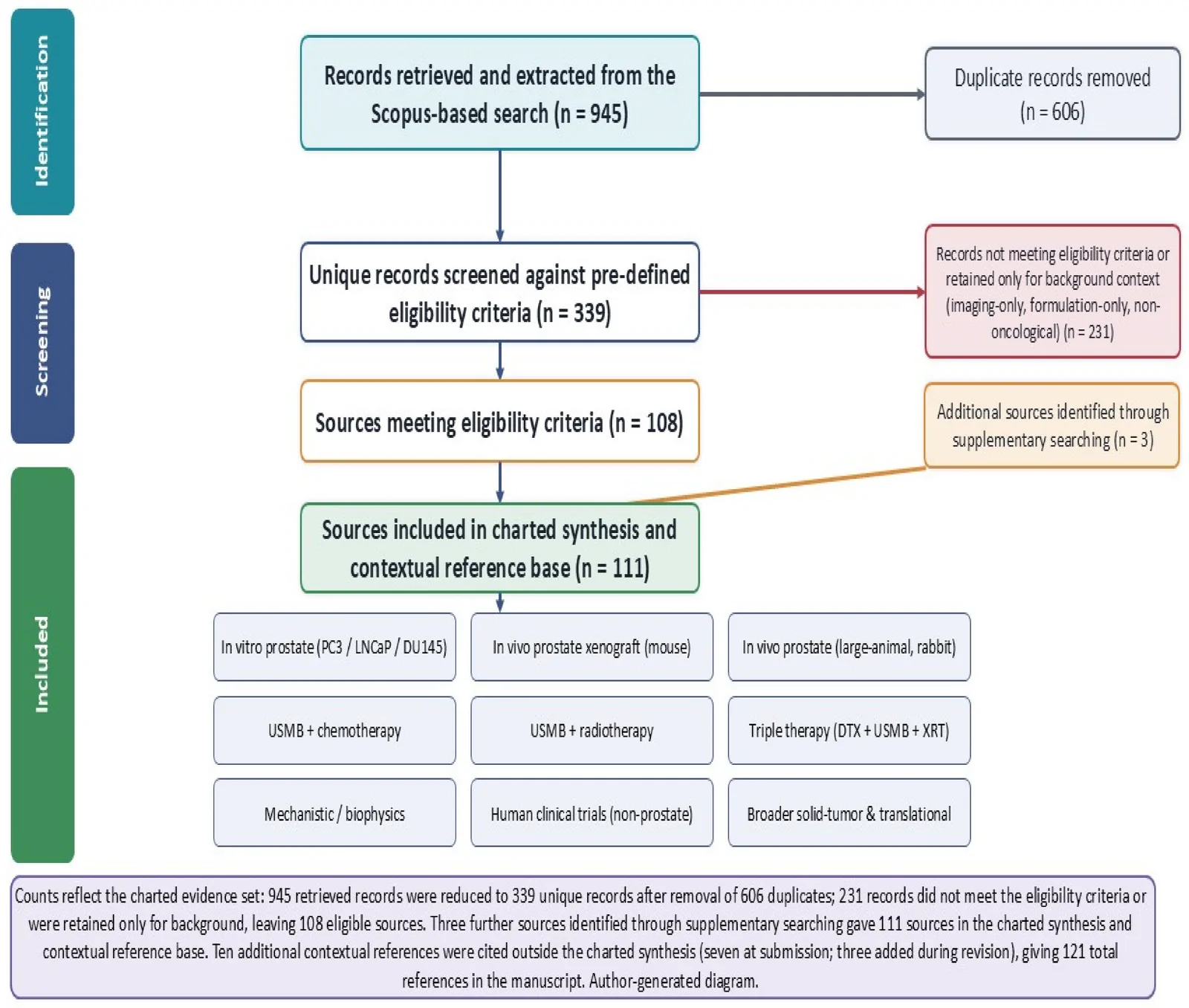
Flow of source identification, screening, and inclusion for this scoping review, informed by PRISMA-ScR reporting principles. Counts reflect the charted evidence set. The structured Scopus search retrieved 945 records; 606 duplicates generated by overlapping search blocks were removed, leaving 339 unique records for screening. Of these, 231 records did not meet the eligibility criteria or were retained only for background context, leaving 108 eligible sources. We included three additional sources identified through supplementary searching, bringing the charted synthesis and contextual reference base to 111 sources; the flow diagram reports these 111 charted sources. The reference list also includes contextual citations that were never charted: seven cited only in the discussion and future-directions sections of the submitted version, and three added during revision [28,29,30], for a total of 121 references in the final list. Author-generated diagram. The executed query string and search details are provided in Supplementary Table S2, and the per-stage charting counts are provided in Supplementary Table S4.

Scopus was the primary bibliographic search database, chosen for its broad coverage of biomedical, oncology, therapeutic-ultrasound, and physical-acoustics literature relevant to this cross-disciplinary review. The structured database query was executed in Scopus and formed the basis of the screening funnel. PubMed/MEDLINE, Crossref, publisher records, and Web of Science/SCIE indexing were used as supporting resources for bibliographic verification, DOI confirmation, source-level checking, and cross-indexing; they were not used as separate queried databases contributing additional records to the screening funnel. The only records entering the funnel beyond the Scopus search were the three supplementary sources identified through reference-list and citation searching. The search combined three concept blocks with the Boolean AND, terms within each block combined with OR, and truncation (the asterisk wildcard) used to capture word variants: (i) the disease ”prostate cancer” OR “prostate neoplasm*” OR “prostate carcinoma” OR PC3 OR “PC-3” OR LNCaP OR DU145; (ii) the modality microbubble* AND (ultrasound OR sonoporation OR sonopermeation OR cavitation OR “ultrasound-stimulated” OR “ultrasound-activated” OR “focused ultrasound”); and (iii) the therapeutic context-docetaxel OR chemotherapy OR radiotherapy OR radiation OR radiosensiti* OR chemo-radio* OR “drug delivery” OR vascular OR apoptosis. To capture mechanistic and translational context, a separate supplementary block relaxed the disease terms to retrieve foundational sonoporation-biophysics, treatment-parameter, and clinical-translation records; we combined results from the two blocks before deduplication. Supplementary Table S2 reproduces the exact executed query string, database field codes, filters, and search details verbatim, which match the terms above. Both executed query strings, the primary block and the supplementary block, are reproduced character for character in Supplementary Table S2.3 and can be pasted directly into the Scopus advanced-search interface so that the search is reproducible by a reader. We conducted the final search on 28 August 2026. We applied no language, publication-date, subject-area, or access-type restrictions at the search stage; we restricted document type to research articles, reviews, and conference papers with retrievable full records. We applied the prioritization of recent (2021–2026) and prostate-specific work described in Section 2.2 at the synthesis stage rather than as a search filter, so the retrieved record set remained complete. Supplementary Table S2.5 itemizes these settings. Reliance on a single primary search database was a deliberate design choice-Scopus offers the broadest combined indexing of the biomedical, oncology, and physical-acoustics journals across which this literature is dispersed-but it carries a known risk that records indexed only in MEDLINE or Embase were missed. We applied two mitigations: we cross-checked retrieved records against Web of Science/SCIE indexing, and we searched the reference lists of included sources, which yielded the three supplementary sources shown in Figure 1; we acknowledge the residual risk of missed studies as a limitation in Section 12. The search was not re-executed after 28 August 2026, so literature published between the search date and the revision of this article was not systematically screened. Three contextual references identified during revision [28,29,30] were added outside the systematic search and are flagged accordingly in Supplementary Table S3; no other post-search studies were incorporated.

### 2.2. Eligibility Criteria

Records were eligible if they reported original experimental data or authoritative review-level synthesis relevant to ultrasound-activated microbubbles as chemo- or radio-enhancers, prioritizing: (i) prostate cancer models (in vitro and in vivo); (ii) combinations of microbubbles with chemotherapy, radiotherapy, or both; and (iii) mechanistic studies of sonoporation, cavitation bioeffects, vascular/endothelial responses, and ceramide signaling that bear directly on those combinations. We included foundational biophysics papers and human clinical trials of ultrasound-microbubble-enhanced chemotherapy or radiotherapy at any tumor site to support mechanism and translation. We excluded records or consulted them only for background without treating them as therapeutic evidence when their primary focus was diagnostic imaging alone, microbubble manufacturing chemistry, or non-oncological applications. Hence, the synthesis remained anchored in cancer therapy rather than drifting into an imaging or engineering survey. No language or date restrictions were applied a priori, although the synthesis prioritized recent work (2021–2026) and prostate-specific studies while retaining foundational older studies where they remain authoritative.

### 2.3. Study Selection and Data Charting

Records retrieved from the database search were exported with title, abstract, and structured extractions of methods, results, and conclusions, then deduplicated. The high duplicate count arose because records were exported from multiple overlapping search blocks-the prostate-specific, mechanistic, and translational queries described in Section 2.1—before deduplication, so that sources retrieved by more than one block appeared repeatedly in the combined export. The records excluded or retained only for background context were predominantly imaging-only, formulation- or manufacturing-only, or non-oncological reports (Figure 1). The structured database search retrieved 945 records. After removal of 606 duplicate records generated by overlapping search blocks, 339 unique records remained for screening. Of these, 231 records did not meet the eligibility criteria or were retained only for background context, leaving 108 eligible sources. Supplementary searching identified three additional sources, bringing the total to 111 sources included in the charted synthesis and contextual reference base.

We use the term “sources” rather than “studies” because the charted set includes original experimental studies alongside review, mechanistic-background, clinical-context, and methodological sources. The 111 sources therefore represent the charted synthesis and contextual reference base, not 111 original experimental studies. Supplementary Table S3 charts experimental and mechanistic evidence sources, whereas review, methodological, and clinical-context references framed the synthesis but were not treated as original therapeutic evidence. The final reference list contains 121 references because seven additional contextual references were cited outside the charted set, and three reviewer-suggested contextual references [28,29,30] were added during revision. These three revision-added references were not part of the systematic search and are flagged as post-search contextual additions in Supplementary Table S3.

The corresponding author screened and initially charted records against pre-defined eligibility criteria. To reduce selection and extraction bias, the second author independently verified the final included-source list, reviewed a targeted subset of exclusion/background-classification decisions, and charted records. The authors resolved any points requiring clarification through discussion. Records whose eligibility was ambiguous-principally those at the boundary between therapeutic evidence and imaging-only or formulation-only work-were resolved by full-text re-reading against the criteria. We did not perform full dual independent screening of all retrieved records, and we acknowledge this as a methodological limitation in Section 12.

Because bibliographic database exports are prone to duplication, incomplete fields, and occasional indexing errors, every source cited here was individually cross-checked against its publisher record, PubMed, or Crossref for bibliographic accuracy, and all quantitative values were taken from the primary reports rather than from secondary summaries. The variables charted for each source were specified as follows: source identifier (first author, year, and DOI); evidence tier; model system and host; cell line; intervention and comparator; ultrasound frequency, acoustic pressure, and pulse or insonation characteristics; microbubble agent and concentration; chemotherapy agent and dose; radiation dose and fractionation; treatment sequencing; reported outcomes (clonogenic survival or viability, apoptosis and necrosis, vascular endpoints, tumor growth and survival, and imaging biomarkers); synergy-assessment method and result where reported; and bibliographic-verification status. These charted data underpin the summative tables presented throughout the review, and the same charting framework is reproduced, with the per-source extraction it produced, in Supplementary Table S3.

### 2.4. Synthesis Approach and Rationale for Not Performing a Meta-Analysis

Evidence was integrated as a narrative mechanistic and translational synthesis structured around the intervention’s biology rather than as a study-by-study catalog. The decision rests specifically on the wide, non-standardized variation in acoustic exposure conditions cataloged study by study in Section 8, where primary reported exposures span roughly 0.24–1.65 MPa-with one parameter-optimization study sweeping 0.05–3.5 MPa and center frequencies 0.5–3 MHz and where pulse duration, pulse-repetition frequency, and insonation time are reported inconsistently or not at all. Because these acoustic variables are the operative determinants of the biological effect (Section 8), pooling effect sizes across such disparate exposures would average together mechanistically distinct interventions rather than estimate a common effect. This exposure heterogeneity compounds further heterogeneity across included studies in model systems (in vitro suspension and adherent cultures, murine and leporine xenografts), microbubble type and concentration, chemotherapy and radiation doses, and most critically in the definition and assay of primary outcomes (clonogenic survival, TUNEL- or ISEL-based (terminal deoxynucleotidyl transferase dUTP nick end labelling; in situ end labelling) cell-death indices, microvessel density, tumor doubling time, and quantitative ultrasound parameters); together with the concentration of the in vivo evidence within a small number of research groups, a quantitative meta-analysis would risk spurious precision and the conflation of mechanistically distinct interventions and was therefore judged inappropriate. Consequently, this review treats heterogeneity as a substantive finding to analyze rather than a nuisance to pool away. Where a validated risk of bias appraisal is required for the animal studies, the SYRCLE tool for preclinical in vivo research would be the appropriate instrument [31]; a formal per-study risk of bias scoring was outside the scope of this mapping synthesis and is noted as a direction for a future systematic review should a sufficiently homogeneous subset accumulate.

## 3. Ultrasound-Activated Microbubbles: Physical Principles and Therapeutic Bioeffects

The therapeutic behavior of a microbubble follows from a simple physical fact: a gas bubble in a sound field is a resonant, highly compressible scatterer, and clinically useful microbubbles happen to resonate in the low megahertz range used for medical ultrasound [24]. At low acoustic pressures, the bubble oscillates gently and near-symmetrically about its equilibrium radius, generating stable, non-inertial microstreaming and shear stresses in the surrounding fluid without necessarily destroying the bubble. As pressure rises, oscillation becomes non-linear and eventually violent: the bubble expands and then collapses in inertial cavitation, emitting a broadband acoustic shock, and near a boundary it collapses asymmetrically, driving a fluid microjet toward the surface [32,33]. These regimes are not merely descriptive categories; they map, at least approximately, onto different biological effects and different acoustic settings, and much of the therapeutic literature can be read as an attempt to place a given application in the right regime.

The forces involved are strongly localized and, at the cellular scale, substantial. Direct measurement of encapsulated-microbubble oscillation alongside membrane permeability has shown that permeabilization of adjacent endothelial cells begins once microbubble-induced shear stress exceeds a threshold on the order of kilopascals [34]. This threshold decreases as the number of oscillation cycles increases and rises roughly linearly with frequency between 0.5 and 2 MHz [34]. High-speed imaging of single collapsing bubbles has directly visualized the microjets and the resulting micron-scale membrane perforations [35], and controlled experiments on single oscillating bubbles against lipid vesicles have confirmed that even linear oscillation, without collapse, can deform and rupture a membrane [36]. The practical implication is that the biological effect can, in principle, be tuned: gentle stable cavitation to permeabilize without killing, or inertial cavitation to disrupt vasculature and provoke cell death.

**Figure 2:**
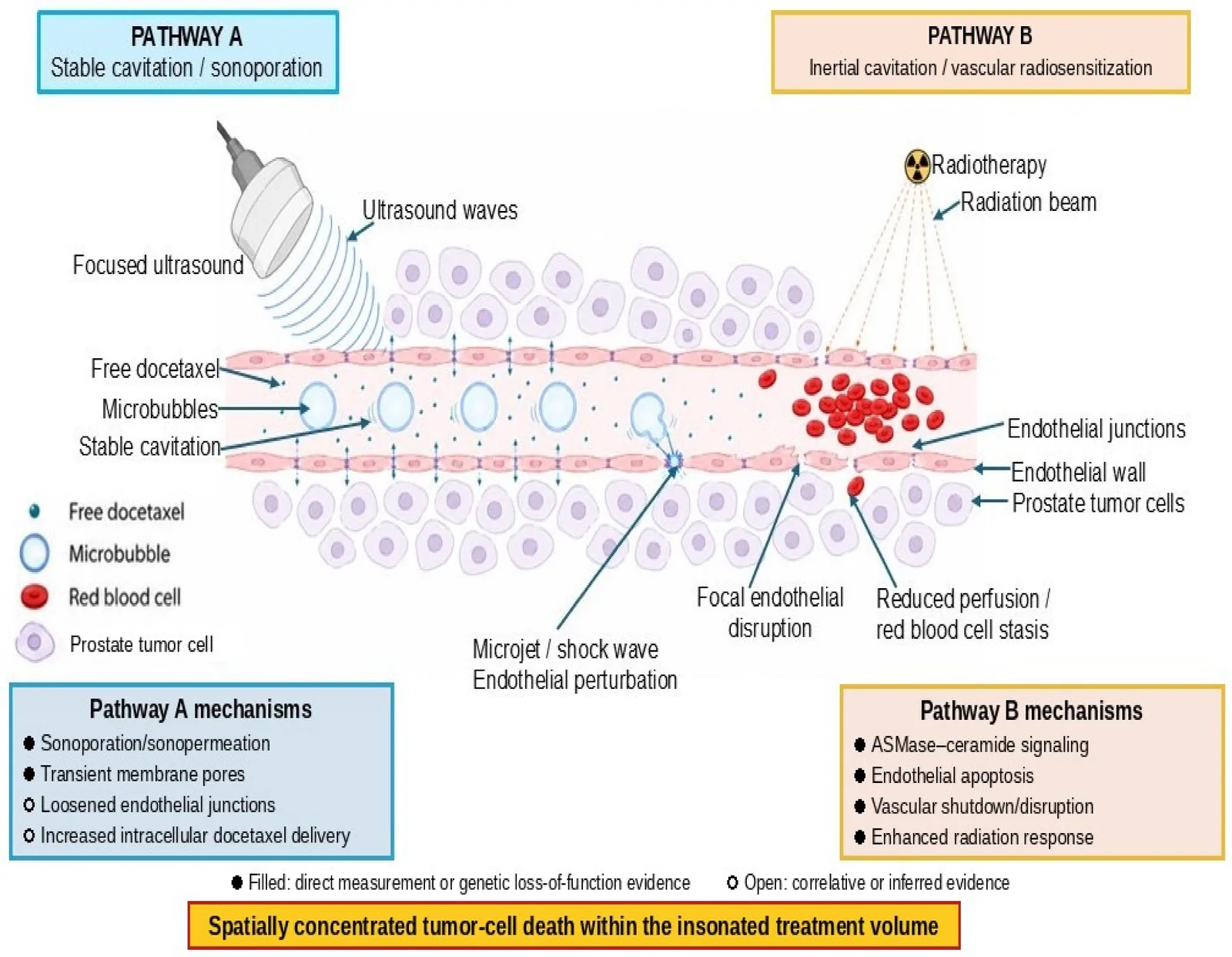
Mechanistic overview of ultrasound-activated microbubbles in prostate cancer chemo-radioenhancement. Focused ultrasound activates intravascular microbubbles within the treatment volume. Stable cavitation may support sonoporation, transient membrane pore formation, loosened endothelial junctions, and increased intracellular delivery of free docetaxel. Inertial cavitation may induce endothelial perturbation, ASMase–ceramide signaling, endothelial apoptosis, vascular shutdown or disruption, and enhanced radiation response. Visual markers distinguish the strength of supporting evidence: filled markers denote mechanisms supported by direct measurement or genetic loss-of-function evidence, and open markers denote mechanisms supported by correlative or inferred evidence in tumor tissue. The schematic summarizes preclinical mechanisms; human prostate efficacy has not been established. Author-generated schematic prepared in Microsoft PowerPoint. The radiation warning symbol is based on the public-domain ionizing-radiation symbol from Wikimedia Commons, and the ultrasound-probe icon is based on a CC0 medical-ultrasound vector icon from SVG Repo. No patient or experimental data are shown.

For an oncology audience, the two therapeutically important consequences of these bioeffects are permeability and vascular perturbation. Increased permeability operates at two levels-across the microvessel wall, enhancing extravasation of circulating drug into the interstitium, and across the plasma membrane of individual cells, enhancing intracellular uptake [25]. Vascular perturbation ranges from a transient increase in perfusion and permeability at low exposures to frank endothelial damage, vasoconstriction, and blood-flow shutdown at higher ones [37]. Which dominates depends on acoustic dose, which is why a single agent can be presented, in different studies, as a drug-delivery vehicle or as an antivascular therapy (Figure 2). Holding both possibilities in view is essential to reading the prostate cancer literature coherently, because chemo-enhancement leans on the former and radio-enhancement, as Section 6 argues, leans substantially on the latter.

Because these distinct processes are too often grouped under a single label, this review defines three operating modes at the outset and refers back to them throughout. In its delivery mode, USMB exploits low-to-moderate-pressure stable cavitation to produce reversible sonoporation and sonopermeation-transient membrane pores, loosened intercellular junctions, and stimulated endocytosis-to increase intracellular and interstitial concentrations of a co-administered drug while preserving cell and vessel viability. In its vascular-disruption mode, USMB uses higher-pressure, longer-pulse exposures in which inertial cavitation perturbs the endothelium and engages ASMase-ceramide signaling, producing endothelial apoptosis and perfusion shutdown that sensitize the tumor to radiation independently of any drug. In its combined mode, exemplified by the triple-therapy studies of Section 7, elements of both are engaged deliberately within one treatment. These modes act on different biological targets, occupy different regions of the acoustic parameter space (Section 8), and fail in different ways; the umbrella term “USMB enhancement” is therefore used in what follows only where a statement genuinely applies across modes, and the operating mode is named wherever the distinction matters.

## 4. Sonoporation and Cavitation-Mediated Drug Delivery

Sonoporation, the transient permeabilization of cell membranes by ultrasound-driven microbubbles, is the mechanistic heart of chemo enhancement, and two decades of work have progressively refined what the term actually denotes. Early studies using ultrasound contrast agents established that insonation opens transient pores that admit otherwise membrane-impermeant molecules, with transfer efficiency falling as molecular size rises: species up to roughly 37 nm cross readily, whereas larger particles enter far fewer cells [38]. Real-time electrophysiology in single cells showed that these pores open with a characteristic delay after insonation and, critically, that membrane resealing depends on extracellular calcium entering through the pore itself [39]. Direct high-speed and confocal imaging subsequently resolved the sequence in endothelial monolayers: a sonoporation event generates pores through both membrane faces that reseal within about two minutes. It can additionally open longer-lived gaps between adjacent cells that persist for tens of minutes [34,40].

However, pore formation is only part of the story. Careful uptake studies have demonstrated that acoustic pressure determines the route of entry: at lower pressures, membrane deformation stimulates endocytosis, whereas at higher pressures frank pores admit molecules directly into the cytosol, and the balance between these subpopulations shifts toward pore-mediated uptake as pressure increases [41]. Endocytosis is an active, ATP-dependent contributor whose importance rises with cargo size [42]. This mechanistic plurality matters therapeutically because the optimal exposure for delivering a small cytotoxic such as docetaxel is not necessarily the optimal exposure for a nanoparticle or a gene construct, and it cautions against uncritically transferring parameters between drug classes. The broader concept of “sonopermeation” was introduced precisely to capture this range of mechanisms-pore formation, junction opening, stimulated endocytosis, and improved perfusion-under one heading [43], and recent syntheses have continued to consolidate the biophysics of sonoporation and the practical determinants of delivery efficiency into a more predictive framework [44,45].

A second lesson from the mechanistic literature is that the consequences of sonoporation outlast the pore. Work in sonoporated cancer cells has shown that even cells that remain immediately viable can suffer delayed disruption of homeostasis, with a substantial fraction undergoing apoptosis or necrosis over the subsequent day and upregulating cytoprotective heat-shock programs in the interim [46]. This is doubly relevant for prostate cancer therapy: it means USMB can contribute directly to cell death rather than acting only as a passive conduit for drug, and it complicates the interpretation of short-term viability assays, since a cell scored as viable at the moment of resealing may be committed to death hours later. In the specific context of the prostate, the same physical process has been shown to loosen the tissue’s diffusion barriers: microbubble-mediated ultrasound increases prostatic permeability in vivo by disrupting endothelial and epithelial tight junctions and downregulating claudin-1, raising local drug concentrations in a way that neither ultrasound nor microbubbles alone achieve [47,48]. That the barrier-opening effect is demonstrable in prostate tissue specifically, and not merely inferred from other organs, is one of the stronger foundations under the chemo-enhancement rationale.

## 5. Microbubble-Mediated Chemotherapy Enhancement in Prostate and Other Solid Tumors

The proposition that USMB can enhance docetaxel activity in prostate cancer rests on a chain of evidence that begins in cell suspension and extends, unevenly, toward the clinic. In vitro, combining ultrasound and microbubbles with docetaxel increases prostate cancer cell kill beyond either agent alone. In an early suspension study of PC3 prostate and MDA-MB-231 breast cells, USMB combined with docetaxel enhanced cytotoxicity in both lines. However, the magnitude was tellingly greater in the chemosensitive breast line than in the more chemoresistant PC3 cells-an early signal that USMB amplifies but does not by itself overcome intrinsic drug resistance [49]. The observation that enhancement is real but bounded by tumor biology recurs throughout the field and is worth holding onto against broader claims.

Two mechanistically distinct strategies for combining USMB with chemotherapy have emerged, and prostate cancer has been a testbed for both. The first exploits sonoporation to increase drug uptake at conventional or low acoustic pressures. The second, “antivascular” strategy uses higher-amplitude exposures to damage tumor vasculature and, in doing so, potentiate a co-administered drug conceptually closer to combining a vascular-disrupting agent with chemotherapy than to enhancing diffusion. In PC3 tumors in mice, weekly antivascular USMB combined with docetaxel produced acute perfusion reduction with enhanced necrosis and apoptosis at 24 h and, over several weeks, significant tumor growth inhibition and prolonged survival that neither docetaxel nor USMB achieved alone [50]. The distinction between uptake-enhancing and antivascular strategies is not merely academic: they call for different acoustic settings, predict different optimal drug ultrasound timing, and may favor different clinical scenarios; conflating them is a common source of confusion in the literature.

The broader solid-tumor experience lends the prostate findings both support and perspective. Sonoporation-enhanced gemcitabine reduced primary tumor burden in orthotopic pancreatic cancer [51], and studies in VX2 tumors have quantified how much the timing of insonation relative to drug infusion matters, with the highest intratumoral drug concentrations achieved when USMB immediately follows chemotherapy [52]. The studies that follow form a distinct evidence stream-payload and construct studies involving doxorubicin, paclitaxel, siRNA, and engineered nanobubbles-and they are summarized here as delivery-platform context; they are deliberately not treated as evidence for docetaxel-specific enhancement, which rests on the studies above and in Section 7. For prostate cancer specifically, targeted and drug-loaded constructs have been explored: microbubble liposome complexes co-delivering doxorubicin and survivin-targeted siRNA reduced LNCaP viability and suppressed survivin in vivo [53]; prostate-specific membrane antigen (PSMA)-targeted, paclitaxel-loaded nanobubbles combined with low-frequency ultrasound inhibited prostate tumor growth and prolonged survival without overt systemic toxicity [54]; and ultrasound-targeted nanobubble destruction increased intratumoral doxorubicin and apoptosis in PC3 xenografts [55], and PSMA-targeted, glutathione-responsive nanoparticles co-delivering docetaxel and enzalutamide have extended the same targeting logic to nanocarrier systems [28]. Related sonoporation approaches have been used to drive tumor-suppressor and gene constructs-wild-type P53 and androgen-receptor-directed siRNA-into prostate cancer cells, broadening the therapeutic repertoire beyond cytotoxics [56,57]. The digestive-oncology experience, where microbubble-assisted chemotherapy has advanced furthest from bench toward clinic, offers a useful template for how such prostate approaches might be developed and where they are likely to stall [58]. Read together, these studies make a reasonably consistent case that USMB increases local drug and payload delivery to prostate tumors; read critically, they are dominated by small xenograft experiments, varied constructs, and short follow-up, and only a minority isolate docetaxel-the agent of greatest clinical relevance-as the partner drug. These payload studies broaden the local-delivery rationale but should not be read as direct evidence that USMB enhances docetaxel specifically under prostate-radiotherapy conditions; that narrower claim rests on the smaller docetaxel-specific literature discussed above and in Section 7.

## 6. Microbubble-Mediated Radiotherapy Enhancement and Radiosensitization

The radioenhancement story is mechanistically distinct from chemo-enhancement, and appreciating that distinction is essential to reading the prostate cancer literature accurately. Whereas sonoporation-based chemo-enhancement is fundamentally about delivery-getting more drug into more cells-USMB radioenhancement, as it has developed, rests substantially on a vascular, endothelial-cell mechanism that does not require any drug at all. The foundational observation came from a prostate model: acoustic stimulation of the tumor vasculature with microbubbles, combined with radiation, enhanced tumor response well beyond radiation alone, through a mechanism implicating rapid endothelial apoptosis rather than direct tumor-cell radiosensitization [26]. The proposed pathway is now reasonably well characterized: microbubble oscillation mechanically perturbs the endothelial membrane, activating acid sphingomyelinase (ASMase), which hydrolyzes membrane sphingomyelin to ceramide; ceramide accumulation drives endothelial apoptosis, and the resulting vascular disruption amplifies radiation-induced tumor cell death [59,60].

This mechanistic account is unusual for the field because it is supported by genetic loss-of-function rather than inferred from correlation. In ASMase-knockout mice, the vascular disruption and the radioenhancement that USMB produces in wild-type animals are largely abolished, and pharmacological manipulation of the sphingolipid pathway modulates the effect in the predicted direction [61,62]. Complementary work has traced the ceramide dependence in endothelial cells directly, showing that interfering with ceramide generation blunts the combined USMB-radiation effect [63] and that manipulating ceramide-metabolizing enzymes such as UGT8 in PC3 cells shifts radiation sensitivity accordingly [64]. Convergent genetic, pharmacological, and imaging evidence pointing to a single pathway is a considerably firmer footing than most proposed mechanisms in ultrasound therapeutics enjoy. It is therefore reasonable to regard the ASMase-ceramide-endothelial axis as the best-supported explanation for USMB radioenhancement, although this pathway may not account for every reported USMB-radiation interaction across tumor types, acoustic settings, and fractionation schedules. However, it is worth being precise about what this mechanism implies. Because the primary target is the endothelium, the effect is expected to scale with the vascular dependence of the tumor and with radiation doses high enough to engage the same ceramide pathway, and it may translate imperfectly to conventionally fractionated low-dose-per-fraction schedules-a tension the field has begun to confront directly (below).

In prostate models specifically, the combination has been reproduced across cell state, dose schedule, and species. Fractionated radiotherapy combined with USMB in PC3 xenografts produced significant vascular disruption and tumor growth reduction over a four-week schedule that neither treatment achieved alone [65], and scaling to a larger, more clinically representative rabbit PC3 model confirmed enhanced vascular shutdown, cell death, and growth delay, with the effect maximized when radiation was fractionated and combined with USMB [66]. Evidence that the mechanism generalizes beyond prostate-USMB radiosensitization has been demonstrated in breast, nasopharyngeal, and oesophageal models, with recurring evidence of vascular disruption and ceramide involvement, strengthening confidence that it reflects a genuine biological principle rather than a peculiarity of one cell line [67,68,69].

Two further observations sharpen this picture rather than merely extending it. First, the radioenhancement effect has been reproduced under magnetic-resonance guidance, which matters less as another positive result than as a demonstration that the treatment can be targeted and monitored with the kind of image guidance a prostate application would demand [70]. Second, and more provocatively, the vascular mechanism is not monolithic. Most USMB radioenhancement is *antivascular-it* sensitizes the tumor by shutting perfusion down-yet a *pro-vascular* strategy that transiently raises tumor perfusion and oxygenation, for instance by co-delivering the reoxygenating agent nitrite, has also been shown to enhance radiation, presumably by relieving the hypoxia that makes tumor cells radioresistant [71]. These are opposite manipulations of the same vasculature, and both improve radiation response. Which is preferable is unlikely to have a single answer: it should depend on the tumor’s baseline perfusion and hypoxic burden, and it is currently unknown which dominates in prostate cancer, where hypoxia is an established driver of radioresistance. This distinction carries real clinical weight. Tumor hypoxia is common in prostate cancer and has been associated with biochemical failure and metastatic progression after radiotherapy [72]. A provascular, reoxygenating strategy is therefore mechanistically aligned with a well-documented driver of local treatment failure, and identifying which patients carry the greatest hypoxic burden may be the key to choosing between antivascular and provascular approaches. In practical terms, such selection could be operationalized with imaging already in clinical or near-clinical use: multiparametric MRI or hypoxia-sensitive imaging before treatment, or CEUS-based perfusion mapping performed in the same session as therapy (Section 10), could stratify tumors into well-perfused lesions-rational candidates for antivascular shutdown-and hypoxic, poorly perfused lesions, for which a provascular, reoxygenating exposure delivered before irradiation is the mechanistically better-matched choice; embedding these perfusion readouts in early trials would allow the stratification hypothesis to be tested rather than assumed. That two contradictory vascular strategies can each claim success is a useful corrective to the assumption that the mechanism is settled and an argument for perfusion- and hypoxia-guided patient selection rather than a single fixed protocol.

At the same time, a recent critical review has highlighted the field’s central unresolved question: although the vascular mechanism is compelling, treatment parameters vary so widely between studies that quantitative comparison is difficult. The interplay between USMB-induced vascular effects and radiation-induced antitumor immunity has barely been examined [73,74]. This dose-per-fraction dependence directly affects which prostate radiotherapy schedule USMB should be paired with, and the field has not yet exploited this alignment. Because the ceramide-mediated endothelial response is engaged most strongly at high doses per fraction-on the order of 8–10 Gy-and indeed the 8 Gy fractions used in most of the preclinical work discussed later in Section 8, the mechanism sits far more naturally with the ultrahypofractionated and stereotactic schedules that have become standard options in localized prostate cancer than with conventional 2 Gy fractions [75]. A first-in-prostate study would therefore better match a stereotactic or focal-boost regimen than conventional fractionation, both because the biology is more likely to be engaged and because the smaller number of fractions makes repeated microbubble administration practical. The mechanism, in short, is well supported in principle but incompletely mapped in the detail that clinical translation will require. It is also worth situating the vascular account within the wider understanding of prostate radioresistance: because hypoxia and perfusion heterogeneity are themselves drivers of radioresistance, a modality that acts directly on tumor vasculature is mechanistically well matched to one of the disease’s core problems, even if that alignment has yet to be exploited deliberately in prostate trials [76,77]. The principal mechanisms and the corresponding strength of evidence are summarized in Table 1.

## 7. Therapeutic Synergy: Docetaxel, Ultrasound-Activated Microbubbles and Radiotherapy in Combination

**Figure 3:**
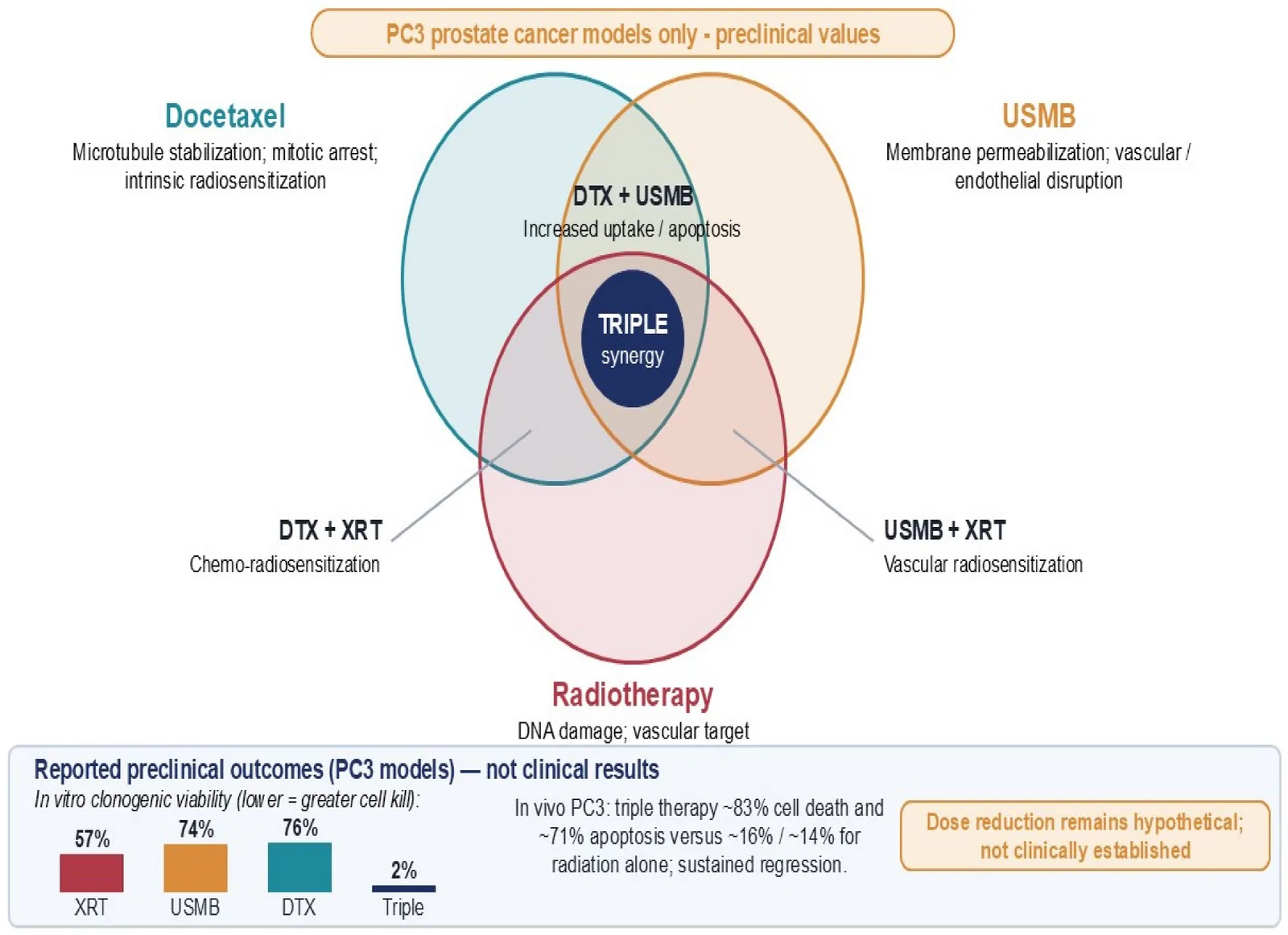
Triple-therapy interaction model summarizing single-, double-, and triple-modality effects reported in PC3 prostate cancer models. Docetaxel, ultrasound-activated microbubbles, and radiotherapy engage partly distinct but overlapping mechanisms, including microtubule stabilization, cavitation-mediated membrane or vascular effects, DNA damage, and vascular radiosensitization. Pairwise combinations may enhance drug uptake, chemo-radiosensitization, or vascular radiosensitization; these pairwise effects were not tested against a formal interaction model and are therefore described as enhancement rather than synergy, whereas the triple combination produced the strongest reported reductions in clonogenic viability and the most pronounced tumor-cell death/apoptosis indices in selected PC3 preclinical models. We demonstrated formal synergy under defined exposure conditions using Bliss independence analysis. All quantitative values shown are preclinical and PC3-model specific, including in vitro clonogenic viability and in vivo PC3 cell-death/apoptosis indices. The proposed widening of the therapeutic window through reduced chemotherapy or radiation dose is presented as a hypothesis and has not been clinically established. Author-generated schematic.

If USMB can enhance chemotherapy through one mechanism and radiotherapy through another, the natural question is whether the three modalities together produce more than the sum of their pairwise effects. The most direct evidence in prostate cancer comes from a pair of linked studies that are, appropriately, central to this review (Figure 3). In vitro, PC3 cells in suspension were exposed to docetaxel, USMB, and radiation alone and in every combination; the triple treatment reduced clonogenic survival to roughly two percent-an order-of-magnitude greater cell kill than any single modality (radiation ~57%, USMB ~74%, docetaxel ~76% viability)-and substantially greater than any pairwise combination, with formal Bliss-independence analysis indicating genuine synergy rather than mere additivity under the conditions tested [81]. Crucially, the synergy was conditional: it depended on docetaxel concentration and exposure time, as well as microbubble concentration, and some double combinations remained additive rather than synergistic. This conditionality is not a weakness of the finding but a central part of it, and it recurs when the work is extended in vivo. Two points of usage should be fixed here. The term “synergy” is reserved in this review for interactions in which a formal analysis, such as Bliss independence, demonstrated supra-additivity; combination benefits not tested against a formal interaction model are described as enhancement or additive benefit. Second, the triple combination is best understood as the deliberate co-engagement of the delivery mode and the vascular-disruption mode defined in Section 3, rather than as a third independent mechanism.

The in vivo companion study carried the triple combination into PC3 xenografts and quantified both cell death and long-term tumor fate. Within the PC3 experimental systems studied, the triple treatment increased tumor cell death and apoptosis roughly five-fold over radiation alone (cell-death and apoptosis indices of approximately 83% and 71% versus 16% and 14%) and, uniquely among the treatment arms, produced sustained tumor shrinkage from the second day onward. In contrast, radiation-treated tumors were only transiently controlled before resuming growth [82]. These effect sizes should be read as specific to selected PC3 models rather than as generalizable quantitative expectations for clinical prostate cancer. The effect sizes are striking, and their internal consistency-histological cell death correlating with a quantitative ultrasound (QUS) spectral parameter across arms-lends them credibility. They should nonetheless be read with the caution the authors themselves invite: the work rests on a single cell line in one immunodeficient xenograft model, follow-up is measured in weeks, and the immunodeficient host removes precisely the antitumor immune contribution that may prove important in patients. The demonstration that triple therapy achieves strong tumor control at relatively low chemotherapy and radiation doses is the most clinically enticing aspect of these results, because dose reduction is the mechanism by which the approach might widen the therapeutic window rather than intensify treatment-but “may permit dose reduction” is a hypothesis generated by these experiments, not a conclusion established by them.

An older but conceptually important line of work supports the plausibility of triple modality combinations from a different direction. MR-guided pulsed focused ultrasound (FUS) was used to enhance docetaxel delivery and combine it with radiotherapy in an orthotopic LNCaP prostate model, with the triple combination producing the greatest tumor growth inhibition among all groups, albeit with the significant caveats of small group sizes, docetaxel-related toxicity, and an inconsistent combination effect in a follow-up experiment [83]. That the earliest and most recent prostate studies reach broadly concordant conclusions through different ultrasound approaches is reassuring; however, both are small, single-model, and short-term, which is a recurring limitation of the evidence base. It is also worth noting a complementary biological rationale outside the ultrasound literature: docetaxel has independent radiosensitizing properties in castration-resistant prostate cancer, linked in recent work to downregulation of caveolin-1, which offers a pharmacological reason to expect chemo-radiotherapy interactions that USMB might then localize and amplify [84].

## 8. Treatment Parameters, Timing, Sequencing, and Optimization

No aspect of USMB therapy is more consequential, or more inconsistently handled across studies, than the exposure parameters. The biological effect is not a fixed property of “ultrasound plus microbubbles” but a sensitive function of acoustic pressure, frequency, pulse duration, pulse-repetition frequency, insonation time, microbubble type and concentration, and the timing of insonation relative to drug and radiation. This parameter sensitivity is simultaneously the source of the approach’s promise-because it can be tuned-and the principal obstacle to comparing studies and to reproducibility.

Foundational in vitro work mapped how permeabilization and viability trade off against acoustic exposure, showing that cell permeability rises and viability falls with increasing peak-negative pressure, pulse-repetition frequency, pulse duration, and insonation time, and identifying a therapeutic-ratio optimum rather than a monotonic “more is better” relationship [85]. The same logic governs the drug-delivery versus antivascular distinction introduced earlier: single-cell and endothelial studies show that short pulses causing limited microbubble displacement favor reversible, viability-preserving sonoporation appropriate for drug delivery, whereas long pulses and higher pressures favor non-displacing, lethal interactions appropriate for antivascular therapy [86]. At the transducer level, a treatment intended to deliver docetaxel and a treatment intended to shut down tumor vasculature are different treatments, and describing both as “USMB” obscures a design decision that determines the outcome.

Although the heterogeneity documented in Table 2 precludes a definitive dose-response synthesis, the charted studies do support an approximate parameter-to-bioeffect orientation. Reversible, viability-preserving sonoporation suited to the delivery mode has generally been reported at frequencies of 0.5–3 MHz with low-to-moderate peak-negative pressures (roughly 0.1–0.6 MPa), short pulses of microseconds to a few tens of cycles, and limited insonation time conditions under which permeabilization is achieved at acceptable viability [41,52,85,86]. Antivascular effects and radioenhancement have generally been produced at higher peak-negative pressures-roughly 0.57–1.65 MPa in the prostate studies charted here-with longer bursts or higher duty cycles and sustained exposures of minutes [50,65,66,87]. These ranges overlap, and frequency, microbubble type and concentration, and tissue context modulate them, so they should be read as orientation rather than prescription. They nonetheless illustrate that the delivery mode and the vascular-disruption mode occupy recognizably different regions of the acoustic parameter space and that a protocol can be designed toward one or the other deliberately rather than discovered by accident.

Microbubble choice compounds the parameter problem. A head-to-head comparison of the three principal clinical agents-SonoVue, Sonazoid, and Optison-found that the best-performing bubble depended on the acoustic conditions, with one agent superior at lower intensities and another at higher, and with marked differences in post-reconstitution stability that have direct practical consequences for reproducible dosing [88]. Microbubble concentration itself can flip a combination from additive to synergistic: in the PC3 triple-therapy work, USMB and radiation became synergistic only above a threshold microbubble concentration [81]. Even an apparently mundane experimental choice-whether cells are treated in suspension or adherent-can determine whether radioenhancement is observed at all, which raises uncomfortable questions about how faithfully suspension experiments predict solid-tumor behavior [89].

Timing and sequencing are the least standardized variables, and the evidence is genuinely mixed. Work on VX2 tumors found the highest intratumoral drug concentrations when USMB immediately followed chemotherapy [52], whereas a prostate xenograft study of USMB-radiation sequencing found the largest tumor effect when microbubbles were delivered around six hours before radiation, consistent with allowing the vascular response to develop. However, the sequencing differences did not reach statistical significance [90]. That two reasonable studies point to different optimal timings for chemo- versus radio-enhancement is exactly what the two-mechanism picture predicts, but it also means no single “correct” sequence carries over to a trial. The optimum for triple therapy-where both mechanisms are in play-remains genuinely unknown. Until exposure conditions and reporting are standardized, the field’s quantitative claims will remain difficult to pool, and this is arguably the single most important methodological reform the area requires. It is worth reframing this heterogeneity as more than an inconvenience. Because the biological effect is genuinely tunable-shifting from viability-preserving drug delivery to lethal antivascular shutdown as pressure and pulse length rise-the parameter space is not noise to be averaged away; rather, it is the operative variable around which a prostate protocol must be built. The goal is not to discover a single “correct” setting but to map which settings produce which biology and then to fix them, deliberately and differently, for a delivery-oriented versus a vascular-oriented indication. Understood this way, the field’s parameter variability is both its central unsolved problem and a direct indication of how to solve it: by treating acoustic dose as a designed therapeutic variable rather than an incidental methodological detail. As a concrete step toward that reform, Supplementary Table S5 proposes a minimum reporting checklist for the acoustic, microbubble, drug, and radiation parameters of USMB chemo- and radioenhancement studies, in the spirit of existing reporting instruments [27,31]; routine adoption of such a checklist would make future studies comparable in precisely the respects that currently frustrate synthesis.

**Table 2:** Reported treatment parameters across representative USMB studies, illustrating the exposure heterogeneity that currently limits cross-study comparison and quantitative pooling. Acoustic, chemotherapy, radiotherapy, microbubble, and model parameters are reported in separate columns. NR denotes not reported or not extractable from the source article, reflecting incomplete reporting in the original work rather than omission by the present author; “-“ denotes not applicable to that study, such as no radiotherapy or no chemotherapy arm. Abbreviations: DTX, docetaxel; XRT, radiotherapy; PNP, peak negative pressure; PRF, pulse-repetition frequency; MB, microbubble; *v*/*v*, volume/volume; fx, fractions. Calibration/derating information is now charted as a separate study-level column.

Study (Ref.)	Frequency	Acoustic Pressure	Pulse/ Insonation	Microbubble (Type, Conc.)	Chemo Agent/Dose	Radiation Dose	Model/Notes	Calibration/ Derating Information
[81]	500 kHz	580 kPa PNP	10 µs pulse; 60 s insonation	Definity, 2% *v*/*v*	DTX, 0.001–0.1 nM	2 Gy	PC3 suspension in vitro	NR
[82]	500 kHz	350 then 580 kPa PNP	16-cycle tone burst (32 µs); 3 kHz PRF; 2 min	Definity, 2 µL/g	DTX, 5 mg/kg	8 Gy single	PC-3 xenograft (mouse); antivascular dual-sequence	NR
[65]	500 kHz	570 kPa PNP	50 ms bursts (3 kHz PRF) repeated every 2 s; 5 min	Definity, 3% *v*/*v*	-	40 Gy/20 fx (2 Gy/day)	PC3 xenograft; USMB 2×/wk × 4 wk	NR
[66]	NR	NR	NR	NR	-	8 Gy single; or 2 Gy ×5/wk × 3 wk (fractionated)	PC3 rabbit xenograft; USMB 2×/wk	NR
[90]	NR	NR	NR	lipid microspheres	-	8 Gy	Prostate xenograft; USMB-XRT sequencing 0–24 h	NR
[50]	1 MHz	1.65 MPa	50 ms bursts (0.00024 duty cycle); 5 min exposure	NR	DTX, 5 mg/kg weekly ×4	-	PC3 xenograft (mouse); antivascular regimen	NR
[85]	500 kHz	570 kPa PNP (0.05–3.5 MPa tested)	8 µs; 3 kHz PRF; 12 s	Definity, 3.3% *v*/*v*	- (model tracer)	-	KHT-C in vitro (parameter-optimization study)	NR
[49]	500 kHz	240 & 580 kPa	30 s insonation; 3 kHz PRF	DA04 (Artenga), 0.5% *v*/*v*	DTX (Taxotere; dose NR)	-	PC3/MDA-MB-231 suspension	NR
[87]	500 kHz	250/570/750 kPa	NR	Definity, 8/80/1000 µL/kg	-	0/2/8 Gy	PC-3 xenograft (mouse)	NR
[52]	3 MHz	0.26 MPa	5-cycle bursts; 0.125% duty cycle	lipid MB	gemcitabine (dose NR)	-	VX2 rabbit (comparator)	NR

Note: NR indicates that the parameter was not reported or could not be reliably extracted from the original study. Calibration/derating information is now reported as a separate study-level parameter where available; where the original study did not report calibration, derating, or related acoustic-field reporting details, the entry is recorded as NR rather than inferred. “-” denotes a parameter not applicable to that study (no chemotherapy or no radiotherapy arm). The prevalence of NR entries highlights incomplete and non-standardized reporting of acoustic and microbubble parameters as a reproducibility barrier in USMB chemo- and radioenhancement studies. Future studies should routinely report acoustic pressure, pulse or burst structure, pulse-repetition frequency, duty cycle, exposure time, microbubble type and concentration, and calibration/derating information, as outlined in Supplementary Table S5.

## 9. Evidence from In Vitro and In Vivo Prostate Cancer Models

Because the PC3 line and its xenografts recur throughout this review, it is worth stepping back to appraise the prostate-specific evidence as a body rather than study by study. PC3 is an androgen-independent, castration-resistant-like human prostate cancer line, which makes it a defensible model for the aggressive, treatment-refractory disease in which better local control is most needed; it is also, however, a single genetic background, and the near-exclusive reliance on it (with occasional LNCaP and DU145 work) is a real external-validity limitation that no amount of internal consistency can remedy. Within that constraint, the prostate literature is more coherent than the broader USMB field. Ultrasound and microbubbles alone induce apoptosis in PC3 cells by downregulating Bcl-2 and upregulating Bax [78]. They can suppress PC3 invasion and migration via reduced matrix-metalloproteinase expression [91], indicating that the technology has direct antitumor effects on prostate cells beyond its role as a delivery adjunct-consistent with the delayed-death phenomenon noted in Section 4.

The genetic identity of PC3 warrants particular emphasis because it limits what these experiments can show. PC3 is PTEN-null and androgen-independent, derived originally from a bone metastasis. It therefore models the aggressive, castration-resistant end of the disease spectrum rather than the androgen-sensitive, localized tumors that constitute the great majority of the radiotherapy-treated population. Because PTEN loss and androgen-receptor status both modulate DNA-damage repair, vascular biology, and intrinsic radiosensitivity, a mechanism such as ASMase-ceramide-mediated endothelial radiosensitization demonstrated predominantly in PC3 cannot be assumed to operate with the same magnitude-or by the same route-in androgen-driven, PTEN-intact prostate cancer. This is a limitation of external validity rather than internal rigor. Still, it is the single most important reason the preclinical signal, however internally consistent, should not be read as a prediction of clinical effect size in the broader human prostate cancer population. Known biology can nonetheless sketch some directions for that caution. PTEN loss constitutively activates PI3K-AKT signaling, which promotes endothelial and tumor-cell survival and antagonizes ceramide-initiated apoptotic signaling.

In contrast, androgen-receptor activity regulates the expression of DNA-damage-repair genes-one basis for the radiosensitizing effect of androgen deprivation-and also shapes prostate vascular biology. PTEN status and androgen-receptor signaling may therefore modify the balance between tumor-cell radiosensitivity and endothelial-mediated radiosensitization, and the magnitude, timing, and route of ASMase-ceramide-mediated effects may differ between PC3 models and androgen-responsive, PTEN-intact prostate cancer models. These are hypotheses rather than established differences, and they are presented here to guide experimental design rather than predict outcomes. Testable predictions follow directly: compare the dose-response of ceramide-dependent endothelial apoptosis between PC3- and LNCaP-class models, and explicitly assess the interaction between androgen deprivation and USMB radioenhancement. Future prostate-specific USMB studies should therefore include androgen-responsive and, where possible, PTEN-intact models alongside PC3 to determine whether sonoporation, vascular disruption, and ASMase-ceramide-mediated radiosensitization are preserved across the molecular diversity of prostate cancer. These are precisely the experiments that the biological-broadening priority of Section 12 calls for.

Layered onto this are the combination studies already discussed: the in vitro and in vivo triple-therapy experiments [81,82], the fractionated-radiation xenograft work [65], and the larger-animal rabbit PC3 model, which begins to address the criticism that mouse xenografts are too small to represent human tumors [66]. The consistent direction of effect across cell state, fractionation schedule, and species is the strongest feature of the prostate evidence. Its weaknesses are equally clear and worth stating plainly. The studies originate from a small number of groups-the in vivo radioenhancement literature in the prostate is dominated by one program-so methods and, to some degree, interpretive assumptions are shared rather than independently replicated, and the apparent consistency of the field partly reflects that common provenance. The reliance on PC3 is near-total, with only occasional LNCaP and DU145 corroboration; because these lines differ in androgen dependence, p53 status, and PTEN loss, effects demonstrated in PC3 cannot be assumed to hold across the molecular heterogeneity of clinical prostate cancer, and the androgen-sensitive disease that dominates the localized, radiotherapy-treated population is barely represented. The near-universal use of immunodeficient xenografts is a particularly consequential gap rather than a generic caveat: the ASMase-ceramide mechanism produces an inflamed, antigen-releasing tumor bed of exactly the kind that radiation-induced antitumor immunity is thought to exploit, so the very models on which USMB radioenhancement rests are those least able to reveal what may prove one of its most important consequences. Taken together, the evidence remains predominantly preclinical, PC3-dominated, and heterogeneous, and the available human studies support feasibility and early safety in selected non-prostate settings without establishing prostate-specific efficacy. Endpoints, finally, are dominated by 24-h histology and weeks-long growth curves rather than durable local control or survival. The prostate evidence is therefore best read as a consistent, mechanistically coherent, but still preliminary proof of concept-strong enough to justify careful clinical development, not strong enough to presume clinical benefit. Representative prostate-specific and comparator preclinical evidence is summarized in Table 3.

## 10. Imaging, Histology, and Response Monitoring

A practical advantage of an ultrasound-based therapy is that the same physics that delivers treatment can, in principle, guide and monitor it, and prostate cancer research has contributed notably to this idea. QUS-the analysis of the raw radiofrequency backscatter rather than the display image-has been developed as a non-invasive readout of treatment-induced cell death. In PC3 xenografts, QUS spectral parameters increased with radiation dose, microbubble concentration, and acoustic pressure and correlated with histological cell death and ceramide staining, offering a label-free surrogate for response [92,93]. The correlation between a QUS mid-band-fit parameter and both cell death and apoptosis in the in vivo triple-therapy study is a concrete example of imaging and biology tracking together across treatment arms [82]. Complementary modalities extend the toolkit: power Doppler and photoacoustic imaging capture the perfusion and oxygenation changes predicted by the vascular mechanism [66], and QUS has also been applied to monitor combined USMB and hyperthermia responses in prostate xenografts [94].

Response monitoring for this therapy matters more than it might first appear, precisely because the effect is so parameter-sensitive and heterogeneous. If treatment success depends on achieving a vascular or permeabilization effect that varies with tumor perfusion, bubble delivery, and acoustic coverage, then a real-time or same-session indicator of whether that effect has been achieved is not a luxury but a plausible prerequisite for reliable delivery. Contrast-enhanced ultrasound (CEUS) already provides perfusion readouts used clinically to confirm treatment effect in USMB-enhanced chemotherapy of other tumors [95], and prostate-directed molecular imaging using PSMA-targeted micro- and nanobubbles points to agents that could unify targeting, treatment, and monitoring [96,97]. Integrated systems capable of performing and imaging microbubble therapy in three dimensions are being built with this convergence in mind [98], and next-generation contrast and phase-shift agents are expanding the theranostic repertoire such systems could draw on [30,99]. The monitoring literature remains largely preclinical, and QUS biomarkers require prospective clinical validation; QUS therefore remains a promising response-monitoring strategy rather than a validated clinical decision tool for prostate USMB treatment. With that caveat, the in-principle ability to see whether the treatment is working is a genuine differentiator of this modality from systemic radiosensitizers.

## 11. Translational Barriers, Safety, and Clinical Feasibility

The gap between the preclinical prostate evidence and clinical application is real but not unbridgeable, and it is narrowing along a route that does not, at present, run through the prostate. The most important translational fact is that USMB-enhanced therapy has already been tested with acceptable early safety in selected patient cohorts for other indications, using commercially available scanners and licensed contrast agents. In inoperable pancreatic cancer, combining gemcitabine with ultrasound and SonoVue was feasible without added toxicity and was associated with more delivered chemotherapy cycles and longer survival than historical controls in a small Phase I study [100,101]; in HER2-negative breast cancer, adding USMB to neoadjuvant chemotherapy was safe and associated with improved local response [95]. Most relevant to the radioenhancement thesis, focused-ultrasound-stimulated microbubbles combined with radiotherapy have now completed early-phase testing: an MR-guided FUS-microbubble radioenhancement trial in breast cancer reported acceptable safety and encouraging tumor responses and durable local control [102,103], a Phase 1 trial extended the same approach to head-and-neck cancer with high short-term response rates [104], and a randomized Phase 2 trial in hepatocellular carcinoma found that adding ultrasound-triggered microbubble destruction to radioembolization improved response and survival with comparable safety [105]. These trials support, at minimum, early clinical deliverability and tolerability in selected non-prostate settings; they do not establish it for the prostate, and the anatomical route to the prostate gland poses delivery problems-acoustic access, target motion, and dosimetric coverage of the whole gland-that the breast and superficial-tumor trials did not have to solve.

The engineering contrast with the completed trials deserves to be stated concretely, because it defines the translational gap. The breast and head-and-neck studies treated superficial targets with wide acoustic windows, minimal respiratory motion, and volumes that a single focused transducer could cover; the liver and pancreatic studies accepted intercostal or transabdominal windows with respiratory management [102,104,105,106]. The prostate presents a different problem in each respect. Acoustic access is transrectal or transperineal, through a narrow window bounded by pelvic bone and the gas-containing rectum; the target moves not with respiration but unpredictably with rectal filling and bladder volume; and whole-gland or hemi-gland coverage requires electronic steering or mechanical scanning from a confined aperture while keeping the rectal wall, urethra, and neurovascular bundles outside the therapeutic focus. None of these problems is insoluble-clinically deployed transrectal therapeutic ultrasound demonstrates that the access route is engineerable-but none of them was solved by the completed non-prostate trials. Those trials therefore establish the feasibility and early safety of USMB-enhanced therapy as a class; they do not establish deliverability, efficacy, or safety in the prostate, and this review treats them strictly as transferable-lessons evidence rather than as prostate evidence.

Safety in the prostate setting likewise deserves a structured statement rather than reassurance by extrapolation. The safety record of diagnostic microbubble imaging cannot be assumed to transfer directly, because therapeutic cavitation regimes use higher pressures, longer exposures, and deliberately bioeffective settings; the relevant question is not whether microbubbles are safe as contrast agents but whether cavitation at therapeutic exposure levels is safe in this anatomy. Four tissue-specific concerns frame that question. The rectal wall lies within millimeters of the posterior gland and directly in a transrectal beam path, and cavitation-mediated endothelial injury there could compound the rectal toxicity that already limits prostate radiotherapy. The urethra traverses the gland, cannot be spared geometrically by any whole-gland exposure, and has essentially uncharacterized tolerance to cavitation. The bladder base and the neurovascular bundles border the target and are implicated in the urinary and erectile toxicities of existing local therapies. Finally, normal prostate parenchyma is the medium through which any whole-gland exposure passes, and microvascular injury there could produce inflammation, edema, or transient obstruction. None of these risks has been quantified at cavitation-level exposures. This argues for the staged development of Section 12: conservative acoustic doses in any first-in-prostate study, focal rather than whole-gland targets, explicit exclusion margins for the rectal wall and urethra, prospective genitourinary and gastrointestinal toxicity endpoints, and same-session monitoring (Section 10) to confirm that bioeffects remain within the intended volume. The porcine-liver experience shows that a parameter space without unacceptable off-target damage can be defined empirically [107]; prostate-specific analogs of that work are a precondition for trials. The available human studies, all conducted outside prostate cancer, are summarized in Table 4.

**Figure 4:**
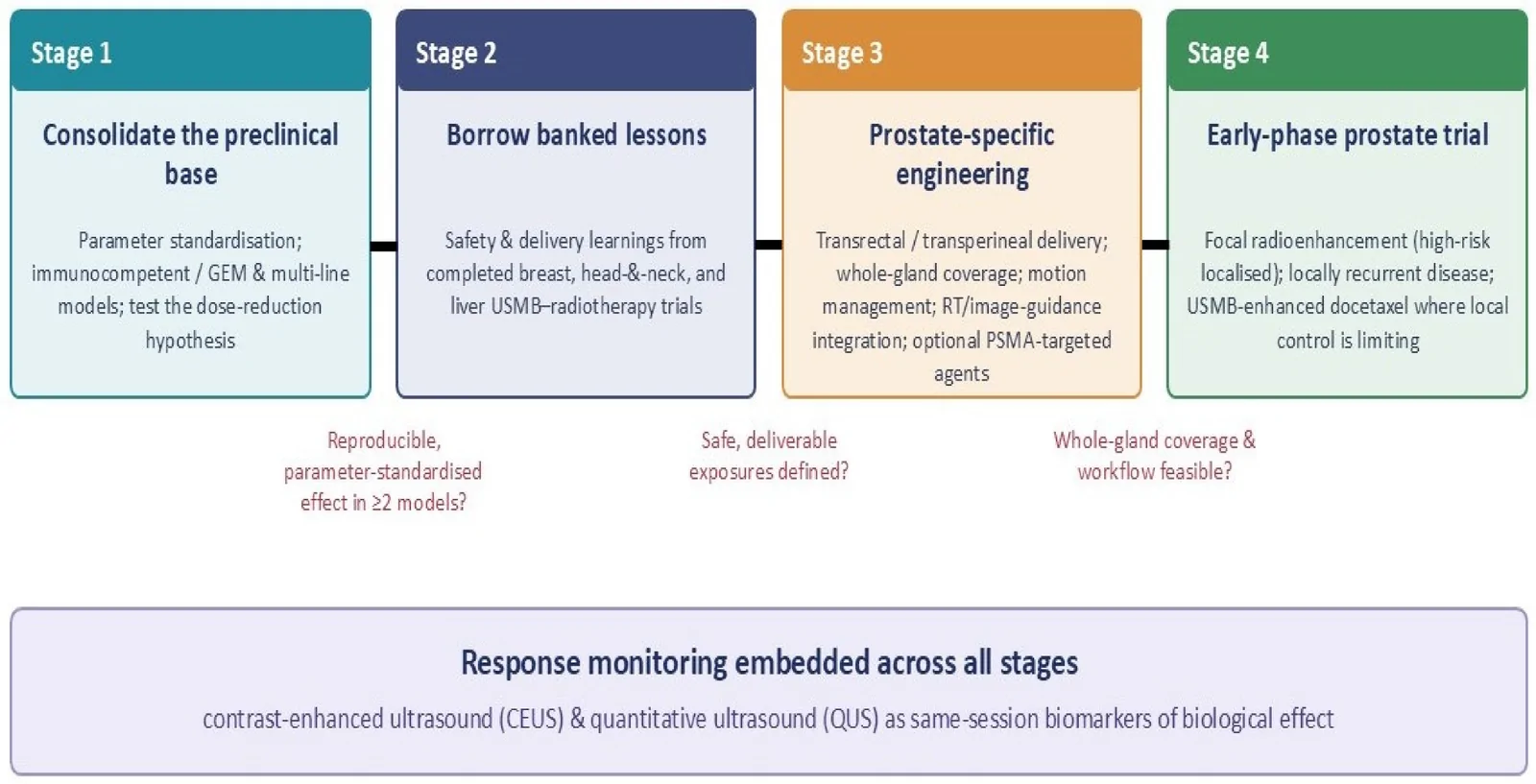
Proposed staged translational roadmap from preclinical prostate cancer models to early-phase prostate trials. Progression is gated by evidence rather than time; standardized and broadened preclinical confirmation should precede adoption of safety and delivery lessons from completed non-prostate USMB-enhanced radiotherapy studies, followed by prostate-specific delivery engineering and cautiously designed early-phase trials. Response monitoring using contrast-enhanced ultrasound (CEUS) and quantitative ultrasound (QUS) should be embedded across all stages as same-session biomarkers of biological effect. The roadmap deliberately positions first-in-prostate testing after, not before, resolution of parameter standardization and prostate-specific delivery feasibility. Author-generated conceptual roadmap.

Several concrete barriers separate the current state from a prostate trial. Parameter standardization, discussed above, is the foremost: without agreed, reproducible exposure conditions, multi-center evaluation is not feasible, and the wide variation in reported settings is a translational liability as much as a scientific one [73]. Microbubble behavior under clinical conditions-reconstitution stability, circulation time, and the difference between imaging-optimized and therapy-optimized agents-needs to be controlled, and manufacturing and regulatory pathways for therapy-intended agents remain less mature than for diagnostic use [23,88,108]. Efforts to place agent design on a rational, reproducible footing-relating shell, size, and gas-core parameters to acoustic response and drug release, and even resolving how the fragments produced when a drug-loaded bubble is destroyed govern uptake-are beginning to provide the engineering vocabulary such standardization will need [109,110,111]. Safety, while reassuring in diagnostic use and in the trials above, must be re-established for the specific pressures and exposure times used therapeutically; dedicated large-animal safety work-for example, in porcine liver-has begun to define the parameter space in which cavitation therapy does not produce unacceptable off-target damage [107]. A workforce and infrastructure dimension is also easy to overlook. Contrast-enhanced ultrasound is governed internationally by multi-society guidance, yet agent approval, equipment provision, and operator training still vary widely between jurisdictions, and national analyses document how far those factors hold uptake below what the guidelines assume [112,113]. A therapy that depends on skilled contrast ultrasound will inherit those constraints. Encouragingly, work showing that a standard clinical ultrasound system can deliver USMB therapy as effectively as a bespoke research rig directly lowers one of these barriers [114]. Credible near-term strategies exist for each of these barriers (Table 5), and their ordering matters. Parameter standardization can begin immediately with consensus reporting (Supplementary Table S5) and shared calibration practices before any new biology is required. Prostate delivery can build directly on transrectal and transperineal platforms already deployed clinically for therapeutic prostate ultrasound and brachytherapy, whose hardware, image registration, and workflow are adaptable to cavitation-mode exposures. Agent variability can be constrained in the near term by standardized preparation and dosing of licensed agents-the approach the completed trials took-while therapy-optimized formulations mature [23,88]. Monitoring is the most tractable barrier, because CEUS and QUS readouts require no hardware beyond the treatment platform itself (Section 10). Sequenced this way-reporting standards first, then delivery engineering on existing platforms, then optimized agents-the list of obstacles becomes an ordered program, which is how Figure 4 presents it.

## 12. Limitations of the Current Evidence and Future Directions

In summary, this literature presents a mechanistically coherent and internally consistent preclinical case, but it is narrower and more preliminary than the volume of publications might suggest. The prostate-specific therapeutic evidence rests heavily on the PC3 model, on a small number of research groups, and on short-term endpoints in immunodeficient hosts; synergy, though formally demonstrated, is conditional on exposure parameters that are not yet standardized; and the single most clinically important claim-that USMB permits equivalent tumor control at reduced chemotherapy and radiation doses, thereby widening the therapeutic window-remains a well-motivated hypothesis rather than a demonstrated result. Because several of the key prostate-specific USMB chemo-radioenhancement studies originate from overlapping research networks, independent replication across laboratories, prostate cancer models, and clinically realistic radiation schedules should be treated as a required step before first-in-prostate efficacy claims are advanced. None of this undermines the approach; it defines the work that remains. Interpreting the field otherwise, or presenting the preclinical effect sizes as if they were clinical ones, would be a disservice to a genuinely promising idea-one best understood, as argued throughout, as a tunable physical sensitization platform whose outcome depends on acoustic dose, vascular state, sequencing, and tumor context rather than as a single fixed technique. Methodologically, the present synthesis is itself limited by the absence of full dual independent screening of all retrieved records-partly mitigated by predefined eligibility criteria, source-level verification, and independent second-author verification of the final included-source list and a targeted subset of exclusion/background-classification decisions and charted records. However, the possibility of missed or differently classified studies cannot be excluded (Section 2.3)-by the absence of prospective registration and by reliance on a single primary search database, the latter partially mitigated by cross-indexing verification and reference-list searching (Section 2.1); its reproducibility was strengthened through explicit eligibility criteria, documented search strings, per-stage counts, and source-level verification.

Three priorities follow directly. The first is standardization: agreed reporting of acoustic pressure, frequency, pulse parameters, insonation time, microbubble type and concentration, and treatment sequencing so that studies can be pooled and a defined treatment can be carried reproducibly into and across clinical centers; the minimum reporting checklist proposed in Supplementary Table S5 is offered as a starting point. The second is biological broadening: immunocompetent and genetically engineered prostate models, additional cell backgrounds beyond PC3, and explicit study of the interaction between USMB-induced vascular effects and antitumor immunity, which current immunodeficient models cannot address and which the wider oncology field increasingly regards as central to radiotherapy response [80,115,116]. The rapidly expanding work on ultrasound-mediated micro- and nanobubble cavitation as a partner for immune-checkpoint blockade and other immunotherapies makes this an unusually timely gap to close [117,118]. The third is prostate-specific translational engineering: solutions for whole-gland acoustic coverage and target motion, integration with existing radiotherapy and image-guidance workflows, and evaluation of whether targeted micro- and nanobubble constructs-including PSMA-directed agents and drug-free cavitation therapies now being explored in prostate models-add enough to justify their regulatory complexity [119,120]. Adjacent mechanistic opportunities, such as using USMB to relieve the elevated interstitial fluid pressure and hypoxia that drive treatment resistance, are biologically attractive and worth testing explicitly in prostate models rather than assumed [76,79].

The immune dimension deserves more than a listed priority, because the mechanism itself predicts immunological consequences in both directions. USMB-induced endothelial apoptosis and perfusion shutdown produce an acutely damaged, antigen-releasing tumor bed; combined with radiation-induced immunogenic cell death, this could plausibly amplify in situ vaccination and the systemic responses that high-dose-per-fraction radiotherapy is hypothesized to promote. The same vascular disruption, however, could equally impair immune-cell trafficking into the tumor, and transient perfusion shutdown may deepen hypoxia-itself immunosuppressive-before any reoxygenation occurs. Which effect dominates likely depends on operating mode, acoustic dose, and timing relative to irradiation, and immunodeficient hosts cannot resolve it. Emerging combination literature shows these questions are now experimentally tractable: perfusion-enhancing USMB potentiates PD-L1 blockade in immunocompetent colon-cancer models [80], and ultrasound-assisted approaches to tumor immunotherapy are developing rapidly [29,117,118]. For prostate cancer-an immunologically “cold” tumor in which checkpoint blockade alone has been largely disappointing-whether USMB-conditioned radiotherapy can shift the tumor microenvironment toward a more immunogenic state is arguably the most important open biological question this platform raises. Immunocompetent prostate models should be designed to answer it explicitly.

A realistic translational roadmap therefore proceeds in stages rather than leaps (Figure 4). It begins by consolidating the preclinical base-standardized parameters, broader and immunocompetent models, and confirmation that the triple-therapy dose-reduction hypothesis holds where it can be tested-then borrows the safety and delivery lessons already banked by the breast, head-and-neck, and liver radioenhancement trials, and only then moves to a cautiously designed early-phase prostate study. Three clinical scenarios align the anatomy, the unmet need, and the mechanism well enough to be plausible entry points, and it is worth being specific about them. The first is focal radioenhancement of a dominant intraprostatic lesion in high-risk localized disease, where the ambition is to raise the biologically effective dose in the lesion while sparing the rectum and urethra-precisely the therapeutic-window problem that motivates the approach-and one already shown to be clinically meaningful, since randomized evidence that an integrated focal boost to the dominant intraprostatic lesion improves biochemical control without added toxicity establishes lesion-directed dose intensification as a validated objective that a vascular radiosensitizer could help achieve [121]. The second is locally recurrent disease after prior radiotherapy, a setting with few good options and a high tolerance for innovative local therapy, where re-irradiation is dose-limited by cumulative normal-tissue exposure and a vascular radiosensitizer could, in principle, allow a lower re-treatment dose. The third is USMB-enhanced docetaxel in diseases where local control is the limiting problem, exploiting sonoporation to raise intratumoral drug and, potentially, to blunt efflux-mediated resistance. In each case, contrast-enhanced or quantitative ultrasound should be embedded from the first patient as a same-session readout of whether the intended vascular or permeabilizing effect was achieved, so a null result can be interpreted as biological absence rather than merely clinical failure. Each scenario also carries a distinct risk profile-normal-tissue dose in the first, cumulative toxicity in the second, and systemic chemotherapy exposure in the third-that should shape trial design and stopping rules. The destination-precise, image-guided, locally intensified chemo-radiotherapy that spares normal tissue is worth the deliberate pace the evidence demands, and overstating current readiness would only jeopardize the careful development it needs.

## 13. Conclusions

Ultrasound-activated microbubbles offer a physically elegant and mechanistically well-founded way to increase the local biological effect of two established prostate cancer treatments within the insonated treatment volume, intending to limit unnecessary systemic or normal-tissue exposure. The evidence assembled here supports two distinct and complementary mechanisms-sonoporation-mediated enhancement of docetaxel uptake and ASMase-ceramide-mediated endothelial radiosensitization-and shows, in prostate models, that docetaxel, microbubbles, and radiation can act together to produce cell kill and tumor control substantially greater than any component alone, under reduced experimental doses in selected models. However, dose reduction remains hypothetical and has not been clinically established. That synthesis is real and encouraging. The practical lesson is that USMB should be treated less as a fixed drug-delivery device and more as a physically tunable sensitizer, configured deliberately by acoustic dose, vascular state, and sequencing for a delivery-oriented or vascular-oriented indication. It is also, at present, preclinical, parameter-sensitive, and built on a narrow experimental base, and the human evidence that provides early support for feasibility and acceptable safety comes from other tumor sites, not the prostate. The task ahead is neither to overstate what has been shown nor to understate its promise, but to do the unglamorous work-standardizing exposures, broadening models, engineering prostate-specific delivery, and designing careful early-phase trials-that would let a compelling laboratory result earn its place in the clinic.

## Figures and Tables

**Table 1:** Proposed mechanisms of USMB-mediated chemo- and radioenhancement in prostate cancer, with an appraisal of the current strength of evidence. “Strong” denotes convergent mechanistic evidence, including loss-of-function data or direct imaging; “moderate” denotes consistent correlative evidence; and “emerging” denotes plausible but limited support. Abbreviations: USMB, ultrasound-activated microbubbles; DTX, docetaxel; RT, radiotherapy; ASMase, acid sphingomyelinase; MMP, matrix metalloproteinase; IFP, interstitial fluid pressure. Symbols: ↑, increase or upregulation; ↓, decrease or downregulation.

Mechanism	Biological Basis	Therapeutic Role	Evidence Strength	Key Refs.
Sonoporation/pore formation	Cavitation-driven transient membrane pores; reseal in <~2 min, Ca^2+^-dependent	↑Intracellular docetaxel/drug uptake	Strong (direct imaging, electrophysiology)	[34,38,39]
Stimulated endocytosis	Membrane deformation/mechanostimulation at lower pressures	↑Uptake of larger cargo; pressure-dependent route	Moderate	[41,42]
Vascular/endothelial permeabilization	Interendothelial gap opening; ↑microvascular permeability	↑Drug extravasation into tumor	Strong	[25,40]
Tight-junction opening (prostate)	↓Claudin-1; disrupted epithelial/endothelial junctions	↑Prostatic drug concentration	Moderate (prostate-specific)	[47,48]
ASMase-ceramide endothelial apoptosis	Mechanotransductive ASMase activation→ ceramide→endothelial apoptosis→vascular shutdown	Radiosensitization via vascular targeting	Strong (genetic loss-of-function)	[26,61,62]
Antivascular perfusion shutdown	High-amplitude inertial cavitation→acute perfusion loss	Potentiates chemo/RT; ↑necrosis	Strong	[37,50]
Provascular reoxygenation	Transient ↑perfusion/oxygenation (e.g., co-delivered nitrite) relieving hypoxia.	Radiosensitization via reoxygenation-opposite direction to antivascular shutdown	Emerging (not tested in prostate)	[71]
Direct pro-apoptotic signaling	↓Bcl-2/↑Bax; delayed post-resealing cell death	USMB as active cytotoxic, not only conduit	Moderate	[46,78]
Hypoxia/interstitial-pressure modulation	Perfusion and IFP changes altering the microenvironment	Possible relief of radioresistance drivers	Emerging	[52,79]
Antitumor immune modulation	Vascular/immune-microenvironment remodeling	Possible cooperation with RT immunity	Emerging (untested in prostate)	[73,80]

**Table 3:** Representative preclinical evidence for ultrasound-activated/stimulated microbubbles (USMB) as chemo- and radioenhancers in prostate cancer, with selected solid-tumor comparators, ordered by evidence tier. The evidence-tier column indicates the level of biological validation, ranging from in vitro evidence to in vivo prostate models, large-animal studies, image-guided models, and genetic mechanistic proof.

Evidence Tier	Model/Cell Line	Intervention	Principal Outcome	Ref.
In vitro (prostate)	PC3 prostate, suspension	USMB + DTX + XRT (2 Gy) vs. single/double	Triple therapy→~2% clonogenic viability vs. 57% (XRT), 74% (USMB), 76% (DTX); Bliss-defined synergy, parameter-dependent	[81]
In vitro (prostate)	PC3/MDA-MB-231 suspension	USMB + DTX, varied pressure and order	Enhanced cell kill; smaller effect in chemoresistant PC3 than in the chemosensitive breast line	[49]
In vitro (prostate)	PC-3 prostate	USMB alone	Apoptosis via ↓Bcl-2/↑Bax; suppressed invasion/migration via ↓MMP-2/MMP-9	[78,91]
In vivo mouse (prostate)	PC-3 xenograft, SCID mice	USMB + DTX + XRT vs. single/double	Cell death 83%/apoptosis 71% vs. 16%/14% (XRT alone); sustained tumor shrinkage from day 2; QUS correlated with cell death	[82]
In vivo mouse (prostate)	PC3 xenograft, mice	Fractionated XRT (40 Gy/20 fx) ± USMB (2×/wk, 4 wk)	Significant tumor shrinkage and vascular disruption (CD68, trichrome, power Doppler) only with combination	[65]
In vivo mouse (prostate)	PC3 xenograft, mice	Antivascular USMB (1 MHz, 1.65 MPa) + DTX (5 mg/kg) weekly ×4	Acute perfusion loss, ↑necrosis/apoptosis at 24 h; tumor shrinkage and prolonged survival vs. monotherapies	[50]
In vivo mouse (prostate)	LNCaP orthotopic mice	MR-guided pulsed FUS + DTX (5 mg/kg) + XRT (4 Gy or 6 Gy single fx)	Greatest tumor growth inhibition with triple therapy; small groups, DTX toxicity, inconsistent combination effect	[83]
In vivo large-animal (prostate)	PC3 xenograft, rabbits	USMB + XRT, single 8 Gy and fractionated	Vascular shutdown, cell death, growth delay; 87% vs. 14% survival at 21 d with fractionated combination	[66]
In vivo mouse (image-guided)	Tumor xenograft, mice	MR-guided USMB + XRT	Independent replication of USMB radioenhancement under MR guidance-relevant to an image-guided prostate workflow	[70]
Mechanistic (genetic)	MCA/129 fibrosarcoma, ASMase^−^/^−^ vs. WT mice	USMB + fractionated XRT	Radioenhancement and vascular disruption abolished in ASMase^−^/^−^→genetic proof of the ceramide pathway.	[61]
Mechanistic (vascular)	Tumor, mice	USMB + nitrite (provascular) + XRT	*Provascular* reoxygenation enhances radiation-opposite vascular direction to anti-vascular shutdown.	[71]
Comparator (solid tumor)	MDA-MB-231 breast xenograft, mice	FUS + microbubbles + XRT (8 Gy)	↑Cell death, ↓vascularity (CD31, Doppler), ↑ceramide, ↑hypoxia	[67]
Comparator (solid tumor)	MIA PaCa-2 orthotopic pancreatic/VX2 rabbit	Sonoporation/USMB + gemcitabine	Reduced tumor burden; highest intratumoral drug concentration when USMB was administered immediately after chemotherapy (2.83× control)	[51,52]

Abbreviations: USMB, ultrasound-activated/stimulated microbubbles; DTX, docetaxel; XRT, radiotherapy; FUS, focused ultrasound; PNP, peak negative pressure; TUNEL, terminal deoxynucleotidyl transferase dUTP nick-end labeling; CD31, cluster of differentiation 31; QUS, quantitative ultrasound; ASMase, acid sphingomyelinase; MMP, matrix metalloproteinase.

**Table 4:** Human clinical studies of USMB-enhanced chemotherapy or radiotherapy. All studies listed were conducted in non-prostate tumors, including pancreatic, breast, head-and-neck, and liver cancers; no human USMB chemo- or radioenhancement study in prostate cancer was identified in the present review. These trials support the broader feasibility and acceptable safety of USMB-enhanced treatment delivery in humans and provide transferable lessons for treatment planning, monitoring, and workflow, but they do not establish prostate-specific efficacy or gland-specific feasibility.

Setting/Phase	Intervention	Key Result	Ref.
Inoperable pancreatic cancer, Phase I (n = 10)	Gemcitabine + USMB (SonoVue, clinical scanner)	No added toxicity; more chemotherapy cycles; median survival 17.6 months versus 8.9 months in historical controls	[100]
HER2-negative breast cancer (n = 10 vs. 16)	NAC + diagnostic USMB (SonoVue)	Smaller post-treatment tumor diameter; more pathologic complete responses; no added toxicity	[95]
Breast cancer, Phase 1 (n = 21; 23 tumors)	MRgFUS-MB + radiotherapy	Safe; 50% complete + 33% partial response at 3 mo; LC 94%/88%/76% at 6/12/24 mo	[102]
Head-and-neck cancer, Phase 1	MRgFUS-MB + radiotherapy	Safe; high 3-month tumor response rates	[104]
Hepatocellular carcinoma, Phase 2 RCT (n = 98)	^90^Y-TARE ± UTMD	Complete/partial response 96% vs. 66% (P = 0.01); improved overall survival (HR 0.51)	[105]
Inoperable pancreatic adenocarcinoma, randomized pilot (n = 19)	Chemotherapy + FUS + microbubbles	Feasible and safe; therapeutic benefit not demonstrated	[106]

Abbreviations: USMB, ultrasound-activated/stimulated microbubbles; NAC, neoadjuvant chemotherapy; LC, local control; MRgFUS-MB, magnetic resonance-guided focused ultrasound with microbubbles; UTMD, ultrasound-triggered microbubble destruction; TARE, transarterial radioembolization.

**Table 5:** Translational barriers to clinical application of USMB chemo-radioenhancement in prostate cancer and candidate solutions.

Barrier	Nature of the Problem	Candidate Solution/ Current Progress	Priority Before First Prostate Trial	Refs.
Parameter non-standardization	Wide variation in pressure, pulse, insonation, and sequencing precludes pooling and reproducibility.	Consensus reporting and standardized exposure protocols; dose-effect mapping	Essential-prerequisite for any reproducible multi-center protocol	[73,85]
Narrow preclinical base	PC3-dominated, few groups, immunodeficient hosts, short endpoints	Immunocompetent/GEM models, additional cell backgrounds, survival endpoints	High-strengthens rationale; can advance in parallel with engineering	[65,82]
Microbubble variability	Agent-dependent performance and post-reconstitution instability	Therapy-optimized, standardized agents; stability-controlled dosing	High-agent and dosing must be fixed before first-in-human use	[23,88]
Prostate delivery/coverage	Whole-gland acoustic access, target motion, dosimetric coverage	Transrectal/transperineal delivery engineering; image guidance; motion management	Essential-gland-specific delivery feasibility is a hard gate	[98]
Safety re-establishment for therapy exposures	Diagnostic safety ≠ therapeutic pressures/times	Large-animal safety studies defining safe parameter space	Essential-required for regulatory and ethics approval	[107]
Regulatory/manufacturing pathway	Therapy-intended agents less mature than diagnostic	GMP manufacturing and regulatory-aligned development	Moderate-can mature alongside early-phase development	[108]
Workflow, training, reimbursement	Contrast ultrasound underused; skills/equipment gaps	Training, integration into RT/oncology workflow; standard clinical scanners shown feasible	Moderate-addressable during controlled roll-out	[113,114]
Response verification	Effect is heterogeneous and parameter-sensitive	CEUS/QUS as same-session biomarkers of biological effect	High-embed from the first patient to interpret null results	[92,95]

Abbreviations: USMB, ultrasound-activated/stimulated microbubbles; GEM, genetically engineered mouse; CEUS, contrast-enhanced ultrasound; QUS, quantitative ultrasound; RT, radiotherapy; GMP, good manufacturing practice.

## Data Availability

The Supplementary Materials provide the PRISMA-ScR checklist, search strategy, per-stage charting counts, and charted data for the principal experimental and mechanistic evidence sources supporting the synthesis.

## Supplementary Materials

The following supporting information can be downloaded at: https://www.mdpi.com/article/10.3390/cancers18183067/s1: Table S1, PRISMA-ScR Checklist; Table S2, Search Strategy, Information Sources, and Database Details (Table S2.1, information sources and the role of each; Table S2.2, concept blocks, terms as executed, and the rationale for each; Table S2.3, executed query strings; Table S2.4, search syntax: field codes, operators, and truncation; Table S2.5, filters and limits applied at the search stage); Table S3, Complete Source Inventory and Study-Level Charted Data (Table S3a, complete inventory of sources cited in this review; Table S3b, per-source charted-data extraction table for the experimental evidence base); Table S4, Records Screened and Per-Stage Charting Counts; Table S5, Proposed Minimum Reporting Checklist for Acoustic, Microbubble, Drug, and Radiation Parameters. All sources listed in the supplementary tables are included in the reference list of the main text and are cited therein; the supplementary tables contain no additional references.
