# Supplementary material for: Ultrasound-Activated Microbubbles for Precision Chemo-Radioenhancement in Prostate Cancer: Sonoporation, Therapeutic Synergy, and Translational Pathways
Source: Cancers (Basel). 2026 Sep 21;18(18):3067. doi: 10.3390/cancers18183067 (PMC13605733; doi:10.3390/cancers18183067)
Supplement: Supplementary file 1 [file cancers-18-03067-s001.zip › cancers-4567372-supplementary.pdf]

Supplementary Materials

Manuscript**:** Ultrasound-Activated Microbubbles for Precision Chemo-Radioenhancement in Prostate Cancer: Sonoporation, Therapeutic Synergy, and Translational Pathways

This document contains Supplementary Tables S1–S5 supporting the scoping-review methodology, source inventory, per-stage charting counts, and proposed minimum reporting checklist for future USMB chemo- and radioenhancement studies. All counts are the charted evidence figures; none were estimated or invented.

Table S1. PRISMA-ScR Checklist

Reporting mapped to the PRISMA-ScR (Preferred Reporting Items for Systematic Reviews and Meta-Analyses extension for Scoping Reviews) checklist (Tricco et al., Ann. Intern. Med. 2018, 169, 467–473). This review was conducted as a scoping review with mechanistic and translational synthesis, informed by PRISMA-ScR reporting principles; it was not prospectively registered, and a single reviewer performed screening.

#	PRISMA-ScR item	Reported?	Location / note
1	Title - identify the report as a scoping review	Yes	Design stated in the Abstract and Section 2 (by journal convention, the review-type label is not placed in the title).
2	Structured summary	Yes	Abstract (Background/Objectives, Methods, Results, Conclusions).
3	Rationale	Yes	Section 1 (Introduction).
4	Objectives	Yes	End of Section 1; Abstract.
5	Protocol and registration	Yes (declared none)	Section 2 - the review was not prospectively registered; stated explicitly.
6	Eligibility criteria	Yes	Section 2.2.
7	Information sources	Yes	Section 2.1 (Scopus; cross-referenced with Web of Science/SCIE; verification against publisher/PubMed/Crossref).
8	Search	Yes	Section 2.1 (concept blocks and wildcards); full executed query, field codes, and search details in Table S2.
9	Selection of sources of evidence	Yes (single-reviewer)	Sections 2.3, 2.4. Screening was performed against pre-defined criteria; dual independent screening was not performed and is not claimed; the mitigation applied is stated in Section 2.3.
10	Data charting process	Yes	Section 2.3; charting fields listed and realised in Table S3.
11	Data items	Yes	Section 2.3; realised in Tables 1–5 and Table S3.
12	Critical appraisal of individual sources	Not performed (justified)	Section 2.4 - formal per-source risk-of-bias scoring was outside the scope of this mapping synthesis; SYRCLE (Hooijmans et al., 2014) is identified as the appropriate tool for a future systematic review of the animal studies. Qualitative evidence-strength appraisal is provided (Table 3 evidence tier; Table 1 evidence strength).
13	Synthesis of results	Yes	Section 2.4 (narrative mechanistic/translational synthesis; explicit rationale for not meta-analyzing).
14	Selection of sources (flow)	Yes	Figure 1 and Table S4 (945 retrieved → 606 duplicates removed → 339 unique → 231 not eligible/background → 108 eligible → +3 supplementary → 111 sources in the charted synthesis and contextual reference base). The submitted reference list contained 118 references: 111 charted/contextual sources plus seven additional contextual references outside the charted set. During peer-review revision, three reviewer-suggested contextual references [28–30] were added outside the systematic search, bringing the final revised reference list to 121 references.
15	Characteristics of sources	Yes	Tables 2, 3, 4; Table S3.
16	Critical appraisal within sources	N/A	See item 12.
17	Results of individual sources	Yes	Tables 2–4; Table S3; Sections 5–10.
18	Synthesis of results	Yes	Sections 3–12.
19	Summary of evidence	Yes	Sections 9, 12–13.
20	Limitations	Yes	Sections 2 (registration/single-reviewer), 8–9, 12.
21	Conclusions	Yes	Section 13.
22	Funding	Yes	Back matter (Funding).

Table S2. Search Strategy, Information Sources, and Database Details

This table reports the complete search specification used for the scoping review. The structured database query was executed in Scopus, which formed the basis of the screening funnel. PubMed/MEDLINE, Crossref, publisher records, and Web of Science/SCIE indexing were used to support cross-indexing, DOI confirmation, bibliographic verification, and source-level checking. No separate query string was executed in these supporting resources, and they contributed no additional records to the screening funnel. Additional sources identified through supplementary searching are reported separately in the source-flow counts. Final search date: 28 August 2026.

Table S2.1. Information sources and the role of each.

Resource	Provider / platform	Role in this review	How it was used
**Scopus**	Elsevier	**Primary search database**	Both executed query blocks (Table S2.3) were run here. All 945 records entering the screening funnel were retrieved and exported from this source. Chosen for its combined coverage of the biomedical, physical-acoustics, and oncology literatures, which this cross-disciplinary topic spans.
**Web of Science Core Collection / Science Citation Index Expanded (SCIE)**	Clarivate	Cross-reference index	Indexing status and bibliographic details of retrieved records were cross-checked where the record was available. No separate query string was executed; this resource contributed no additional records.
**PubMed / MEDLINE**	US National Library of Medicine	Bibliographic verification	Author list, article title, journal, year, volume, issue, and pagination of each cited source were checked against the MEDLINE record.
**Crossref**	Crossref (REST API and web interface)	DOI and metadata verification	Every DOI in the final reference list was resolved and its registered metadata confirmed against the citation as printed.
**Publisher records (journal websites)**	Individual publishers	Primary-source verification	Full text was consulted for every charted source. All quantitative values reported in Tables 2–5 and Table S3b were taken from the primary report rather than from any secondary summary or database abstract.
**Reference-list and citation searching**	-	Supplementary searching	Reference lists of included sources and forward citations were examined after the main screen. This step identified the three additional sources recorded in Figure 1 and Table S4.

We searched only the first resource using a query string. We state the distinction between databases searched and resources used for verification because the two are frequently conflated; the record counts in Figure 1 and Table S4 come from Scopus alone, plus the three supplementary sources.

Table S2.2. Concept blocks, terms as executed, and the rationale for each.

Block	Concept	Terms as executed	Rationale
1	Disease and model	"prostate cancer" OR "prostate neoplasm*" OR "prostate carcinoma" OR PC3 OR "PC-3" OR LNCaP OR DU145	Captures the clinical entity together with the cell lines that dominate the preclinical literature, so that model-based reports indexed without a disease term are still retrieved. Both PC3 and "PC-3" are included because the two spellings are used interchangeably across journals.
2	Intervention and modality	microbubble* AND (ultrasound OR sonoporation OR sonopermeation OR cavitation OR "ultrasound-stimulated" OR "ultrasound-activated" OR "focused ultrasound")	Requires a microbubble term in every retrieved record and pairs it with the acoustic-activation vocabulary, which is applied inconsistently across the acoustics and oncology literatures.
3	Therapeutic context and outcome	docetaxel OR chemotherapy OR radiotherapy OR radiation OR radiosensiti* OR chemo-radio* OR "drug delivery" OR vascular OR apoptosis	Restricts retrieval to therapeutic applications and their principal mechanisms, so that purely diagnostic-imaging reports are not returned.
Supplementary	Mechanism and translation, disease block relaxed	See the supplementary string in Table S2.3	Retrieves foundational sonoporation-biophysics, exposure-parameter, and clinical-translation records that predate the prostate literature or were published outside it, and which the disease-restricted primary block would exclude.

Table S2.3. Executed query strings. Query strings are reproduced verbatim as executed in the Scopus advanced-search interface. The three primary concept blocks were combined with AND; terms within each block were combined with OR. Supporting sources used for verification and cross-checking are described in Table S2.1.

Primary block:

```
TITLE-ABS-KEY(
 ( "prostate cancer" OR "prostate neoplasm*" OR "prostate carcinoma" OR PC3 OR "PC-3" OR LNCaP OR DU145 )
 AND
 ( microbubble* AND ( ultrasound OR sonoporation OR sonopermeation OR cavitation
    OR "ultrasound-stimulated" OR "ultrasound-activated" OR "focused ultrasound" ) )
 AND
 ( docetaxel OR chemotherapy OR radiotherapy OR radiation OR radiosensiti*
    OR chemo-radio* OR "drug delivery" OR vascular OR apoptosis )
)
```

Supplementary block (run separately; results were combined with the primary block before de-duplication):

```
TITLE-ABS-KEY(
 ( microbubble* OR "ultrasound contrast agent*" )
 AND ( sonoporation OR sonopermeation OR cavitation OR "acid sphingomyelinase"
     OR ceramide OR "vascular disruption" OR radiosensiti* )
 AND ( "drug delivery" OR radiotherapy OR radiation OR chemotherapy OR clinical OR trial )
)
```

Table S2.4. Search syntax: field codes, operators, and truncation.

Element	Syntax as used	Function
Field code	`TITLE-ABS-KEY( )`	Restricts matching to the title, abstract, and author keywords of a record. Full-text and cited-reference matches are therefore excluded.
Boolean AND	Between concept blocks	Every retrieved record satisfies all blocks simultaneously.
Boolean OR	Between terms within a block	Any single term within a block satisfies that block.
Truncation (wildcard)	Trailing asterisk, e.g. `microbubble*`	Retrieves all word endings from the stem, so that singular, plural, and compound forms are captured in one term.
Phrase searching	Double quotation marks, e.g. `"focused ultrasound"`	Forces adjacent-word matching so that the two words are retrieved only as a phrase.
Grouping	Parentheses	Sets the order of Boolean evaluation, so that each block is resolved before the blocks are combined.

Truncation operators used (reproduced unescaped): microbubble*, radiosensiti*, chemo-radio*, prostate neoplasm*, ultrasound contrast agent*.

Table S2.5. Filters and limits applied at the search stage.

Parameter	Setting
Language	No restriction applied.
Publication date	No restriction applied. Foundational older studies were retained where they remain the authoritative source for a mechanism or parameter.
Document type	Restricted to research articles, reviews, and conference papers with retrievable full records.
Subject area	No restriction applied, so that acoustics, engineering, and oncology records were retrieved together.
Access type	No restriction applied.
Prioritisation of recent and prostate-specific work	Applied at the synthesis stage (2021–2026 and prostate-specific studies prioritized), not as a search filter, so that the retrieved set remained complete.

Record export and de-duplication. Records were exported with title, abstract, and a structured extraction of methods, results, and conclusions. The primary and supplementary blocks were combined before de-duplication. Because the two blocks overlap substantially by design, 606 of the 945 retrieved records were duplicates; the per-stage counts are given in Table S4 and shown in Figure 1.

Reproducibility. Both query strings above are reproduced exactly as executed and can be pasted directly into the Scopus advanced-search interface. Because Scopus is updated continuously, re-running the strings after the final search date will return a larger record set than the one reported here.

Table S3. Complete Source Inventory and Study-Level Charted Data

Table S3a. Complete inventory of sources cited in this review (n = 121)**.** Every source in the final reference list is listed, in reference-number order, with the role it plays in the review and the section(s), manuscript table(s), and supplementary table in which it is used. Reference numbers correspond exactly to the numbered reference list of the main manuscript. The 111 sources shown in the Figure 1 flow diagram are those charted as therapeutic evidence or as the contextual reference base; the remaining seven, marked †, were cited only in the discussion and future-directions sections and were not charted - reporting-guidance and risk-of-bias instruments (refs 27,31), the prostate radiotherapy guideline and focal-boost trial (refs 3,121), international contrast-enhanced-ultrasound guidance (ref 112), and hypoxia and fractionation context (refs 72,75). "Source type" records the study design reported by the source itself. Study-level acoustic, microbubble, drug, and radiation parameters are charted in Table S3b for the experimental sources that form the primary evidence base; such parameters were not extracted for review, guideline, methodological, and clinical-context sources, which do not report them. DOIs are live links. Three contextual references added during peer-review revision (refs 28–30, marked *) are included in the inventory; they were identified through the review process rather than the systematic search and are not charted as therapeutic evidence.

No.	Source	Title	Source type	Role in the review	Cited in	DOI
1	Philippou et al., 2020	Harnessing the potential of multimodal radiotherapy in prostate cancer	Review	Prostate radiotherapy: multimodal treatment context	§1	10.1038/s41585-020-0310-3
2	Safavi et al., 2023	Late Toxicities of Prostate Radiotherapy: Can We Further SMARTen Up the Therapeutic Index?	Review	Late radiotherapy toxicity and the therapeutic index	§1	10.1016/j.euf.2023.03.020
3	Cornford et al., 2024	EAU-EANM-ESTRO-ESUR-ISUP-SIOG Guidelines on Prostate Cancer-2024 Update. Part I: Screening, Diagnosis, and Local Treatment with Curative Intent	Guideline	EAU-EANM-ESTRO-ESUR-ISUP-SIOG prostate cancer guideline	§1 †	10.1016/j.eururo.2024.03.027
4	Kim et al., 2023	Dose-escalated radiotherapy for clinically localized and locally advanced prostate cancer	Systematic review / meta-analysis	Dose-escalated radiotherapy: benefit and toxicity ceiling	§1	10.1002/14651858.cd012817.pub2
5	Wu et al., 2023	Molecular mechanisms of tumor resistance to radiotherapy	Review	Molecular mechanisms of radioresistance	§1	10.1186/s12943-023-01801-2
6	Sosa et al., 2026	Cellular signaling and radioresistance in prostate cancer	Review	Signalling pathways in prostate radioresistance	§1	10.1016/j.cellsig.2026.112367
7	Longoni et al., 2025	Biochemical recurrence after radical prostatectomy and postoperative radiotherapy: current evidence and controversial issues	Review	Biochemical recurrence after prostatectomy and radiotherapy	§1	10.1097/mou.0000000000001292
8	Rahman et al., 2024	Management strategies for radio-recurrent prostate cancer: a comprehensive review	Review	Management of radio-recurrent prostate cancer	§1	10.21037/tcr-24-245
9	Li et al., 2025	Targets for improving prostate tumor response to radiotherapy	Review	Pharmacological targets for prostate radiosensitization	§1	10.1016/j.ejphar.2024.177149
10	Matos et al., 2026	Overcoming radioresistance in prostate cancer by targeting DNA damage repair	Review	Targeting DNA-damage repair to overcome radioresistance	§1	10.1016/j.canlet.2025.218183
11	Tannock et al., 2004	Docetaxel plus Prednisone or Mitoxantrone plus Prednisone for Advanced Prostate Cancer	Randomised clinical trial	Docetaxel survival benefit in mCRPC (TAX 327)	§1	10.1056/nejmoa040720
12	Berthold et al., 2008	Docetaxel Plus Prednisone or Mitoxantrone Plus Prednisone for Advanced Prostate Cancer: Updated Survival in the TAX 327 Study	Clinical trial (updated analysis)	TAX 327 updated survival	§1	10.1200/jco.2007.12.4008
13	Sweeney et al., 2015	Chemohormonal Therapy in Metastatic Hormone-Sensitive Prostate Cancer	Randomised clinical trial	Docetaxel in hormone-sensitive disease (CHAARTED)	§1	10.1056/nejmoa1503747
14	James et al., 2016	Addition of docetaxel, zoledronic acid, or both to first-line long-term hormone therapy in prostate cancer (STAMPEDE): survival results from an adaptive, multiarm, multistage, platform randomized controlled trial	Randomised clinical trial	Docetaxel added to hormone therapy (STAMPEDE)	§1	10.1016/s0140-6736(15)01037-5

No.	Source	Title	Source type	Role in the review	Cited in	DOI
**15**	Sekino et al., 2020	Molecular mechanisms of docetaxel resistance in prostate cancer	Review	Molecular mechanisms of docetaxel resistance	§1	10.20517/cdr.2020.37
**16**	Matsukawa et al., 2025	Toxicities of taxane-based chemotherapy in prostate cancer	Review	Toxicity of taxane-based chemotherapy	§1	10.1097/mou.0000000000001296
**17**	Linke et al., 2022	Comprehensive Evaluation of Multiple Approaches Targeting ABCB1 to Resensitize Docetaxel-Resistant Prostate Cancer Cell Lines	Original study (in vitro)	ABCB1-targeted resensitization of docetaxel-resistant prostate lines	§1	10.3390/ijms24010666
**18**	Xing et al., 2026	RIPK2 induces docetaxel resistance in prostate cancer through the NF-kB/P-gp signaling pathway	Original study (preclinical)	RIPK2–NF-κB/P-gp axis in docetaxel resistance	§1	10.1371/journal.pone.0341445
**19**	Sun et al., 2024	NOTCH3 promotes docetaxel resistance of prostate cancer cells through regulating TUBB3 and MAPK signaling pathway	Original study (preclinical)	NOTCH3/TUBB3 signalling in docetaxel resistance	§1	10.1111/cas.16040
**20**	Rizzo, 2021	Mechanisms of docetaxel resistance in prostate cancer: The key role played by miRNAs	Review	miRNA networks in docetaxel resistance	§1	10.1016/j.bbcan.2020.188481
**21**	Hatano et al., 2023	Systemic Therapies for Metastatic Castration-Resistant Prostate Cancer: An Updated Review	Review	Systemic therapy landscape in mCRPC	§1	10.5534/wjmh.220200
**22**	Ng et al., 2026	Treatment Strategies in Metastatic Castration-Resistant Prostate Cancer: A Narrative Review of Considerations in the Post-ARTA, Post-Docetaxel Setting	Review	Post-ARTA, post-docetaxel treatment strategies	§1	10.1007/s40487-025-00412-9
**23**	Stride et al., 2020	Microbubble Agents: New Directions	Review	Microbubble agents: composition and new directions	§1, 11; Table 5	10.1016/j.ultrasmedbio.2020.01.027
**24**	Kooiman et al., 2014	Acoustic behavior of microbubbles and implications for drug delivery	Review	Acoustic behaviour of microbubbles and drug-delivery implications	§1, 3	10.1016/j.addr.2014.03.003
**25**	Chowdhury et al., 2020	Ultrasound and microbubble mediated therapeutic delivery: Underlying mechanisms and future outlook	Review	Mechanisms of USMB-mediated therapeutic delivery	§1, 3; Table 1	10.1016/j.jconrel.2020.06.008
**26**	Czarnota et al., 2012	Tumor radiation response enhancement by acoustical stimulation of the vasculature	Original study (preclinical)	Foundational demonstration of vascular USMB radioenhancement	§1, 6; Table 1	10.1073/pnas.1200053109
**27**	Tricco et al., 2018	PRISMA Extension for Scoping Reviews (PRISMA-ScR): Checklist and Explanation	Methodological guidance	PRISMA-ScR reporting principles applied to this review	§2 †	10.7326/m18-0850
28	Chen et al., 2022	Prostate-specific membrane antigen targeted, glutathione-sensitive nanoparticles loaded with docetaxel and enzalutamide for the delivery to prostate cancer	Original study (preclinical, prostate)	PSMA-targeted docetaxel nanocarrier context *	§5	10.1080/10717544.2022.2110998
29	Fan et al., 2026	Research Progress on the Application of Ultrasound Assistance in Tumor Immunotherapy	Review	Ultrasound-assisted tumor immunotherapy context *	§12	10.2174/0109298673337725250108091641
30	Han et al., 2024	The advance of ultrasound-enabled diagnostics and therapeutics	Review	Ultrasound theranostics context *	§10	10.1016/j.jconrel.2024.08.039
**31**	Hooijmans et al., 2014	SYRCLE's risk of bias tool for animal studies	Methodological guidance	SYRCLE risk-of-bias framework for animal studies	§2.4 †	10.1186/1471-2288-14-43
**32**	Collis et al., 2010	Cavitation microstreaming and stress fields created by microbubbles	Original study (biophysics)	Cavitation microstreaming and shear stress fields	§3	10.1016/j.ultras.2009.10.002

No.	Source	Title	Source type	Role in the review	Cited in	DOI
**33**	Prentice et al., 2005	Membrane disruption by optically controlled microbubble cavitation	Original study (biophysics)	Membrane disruption by controlled microbubble cavitation	§3	10.1038/nphys148
**34**	Helfield et al., 2016	Biophysical insight into mechanisms of sonoporation	Original study (biophysics)	Biophysical mechanism of sonoporation	§3, 4; Table 1	10.1073/pnas.1606915113
**35**	Kudo et al., 2009	Sonoporation by Single-Shot Pulsed Ultrasound with Microbubbles Adjacent to Cells	Original study (biophysics)	Single-shot pulsed sonoporation adjacent to cells	§3	10.1016/j.bpj.2009.02.072
**36**	Marmottant et al., 2003	Controlled vesicle deformation and lysis by single oscillating bubbles	Original study (biophysics)	Vesicle deformation and lysis by oscillating bubbles	§3	10.1038/nature01613
**37**	El Kaffas et al., 2018	Tumour Vascular Shutdown and Cell Death Following Ultrasound-Microbubble Enhanced Radiation Therapy	Original study (preclinical)	Vascular shutdown and cell death after USMB-enhanced radiation	§3; Table 1	10.7150/thno.19010
**38**	Mehier-Humbert et al., 2005	Plasma membrane poration induced by ultrasound exposure: Implication for drug delivery	Original study (in vitro)	Plasma-membrane poration and drug-delivery implications	§4; Table 1	10.1016/j.jconrel.2005.01.007
**39**	Deng et al., 2004	Ultrasound-induced cell membrane porosity	Original study (in vitro)	Quantification of ultrasound-induced membrane porosity	§4; Table 1	10.1016/j.ultrasmedbio.2004.01.005
**40**	Beekers et al., 2020	Opening of endothelial cell-cell contacts due to sonoporation	Original study (in vitro)	Opening of endothelial cell–cell contacts by sonoporation	§4; Table 1	10.1016/j.jconrel.2020.03.038
**41**	De Cock et al., 2015	Ultrasound and microbubble mediated drug delivery: Acoustic pressure as determinant for uptake via membrane pores or endocytosis	Original study (in vitro)	Acoustic pressure determines pore versus endocytic uptake	§4; Table 1	10.1016/j.jconrel.2014.10.031
**42**	Meijering et al., 2009	Ultrasound and Microbubble-Targeted Delivery of Macromolecules Is Regulated by Induction of Endocytosis and Pore Formation	Original study (in vitro)	Endocytosis and pore formation in macromolecule delivery	§4; Table 1	10.1161/circresaha.108.183806
**43**	Snipstad et al., 2018	Sonopermeation to improve drug delivery to tumors: from fundamental understanding to clinical translation	Review	Sonopermeation from fundamentals to clinical translation	§4	10.1080/17425247.2018.1547279
**44**	Tu et al., 2022	Ultrasound-Mediated Drug Delivery: Sonoporation Mechanisms, Biophysics, and Critical Factors	Review	Sonoporation mechanisms, biophysics, and critical factors	§4	10.34133/2022/9807347
**45**	Bouakaz et al., 2024	From concept to early clinical trials: 30 years of microbubble-based ultrasound-mediated drug delivery research	Review	Thirty years of USMB drug-delivery research	§4	10.1016/j.addr.2024.115199
**46**	Duan et al., 2021	Sonoporation generates downstream cellular impact after membrane resealing	Original study (in vitro)	Downstream cellular effects after membrane resealing	§4; Table 1	10.1038/s41598-021-84341-3
**47**	Shang et al., 2015	The Roles of Tight Junctions and Claudin-1 in the Microbubble-Mediated Ultrasound-Induced Enhancement of Drug Concentrations in Rat Prostate	Original study (in vivo)	Tight junctions and claudin-1 in rat prostate permeability	§4; Table 1	10.1007/s00232-015-9834-5
**48**	Li et al., 2012	Mechanisms of Prostate Permeability Triggered by Microbubble-Mediated Acoustic Cavitation	Original study (preclinical, prostate)	Mechanisms of cavitation-triggered prostate permeability	§4; Table 1	10.1007/s12013-012-9383-9
**49**	Karshafian et al., 2010	Enhancing chemotherapy by ultrasound and microbubbles: Effect of acoustic pressure and treatment order in in vitro suspension of breast and prostate cancer cells	Original study (in vitro) - conference proceeding	USMB + docetaxel: acoustic pressure and treatment order (prostate, breast)	§5; Table 2, 3; Table S3b	10.1109/ultsym.2010.5935914
**50**	Goertz et al., 2012	Antitumor Effects of Combining Docetaxel (Taxotere) with the Antivascular Action of Ultrasound Stimulated Microbubbles	Original study (in vivo, prostate)	Antivascular USMB combined with docetaxel	§5; Table 1, 2, 3; Table S3b	10.1371/journal.pone.0052307

No.	Source	Title	Source type	Role in the review	Cited in	DOI
**51**	Kotopoulis et al., 2014	Sonoporation-Enhanced Chemotherapy Significantly Reduces Primary Tumour Burden in an Orthotopic Pancreatic Cancer Xenograft	Original study (in vivo, pancreatic)	Sonoporation-enhanced gemcitabine, orthotopic model	§5; Table 3; Table S3b	10.1007/s11307-013-0672-5
**52**	Luo et al., 2022	Optimal treatment occasion for ultrasound stimulated microbubbles in promoting gemcitabine delivery to VX2 tumors	Original study (in vivo, VX2)	Timing of USMB relative to gemcitabine delivery	§5, 8; Table 1, 2, 3; Table S3b	10.1080/10717544.2022.2115163
**53**	Bae et al., 2016	Ultrasound-Guided Delivery of siRNA and a Chemotherapeutic Drug by Using Microbubble Complexes: In Vitro and In Vivo Evaluations in a Prostate Cancer Model	Original study (preclinical, prostate)	Microbubble complexes co-delivering siRNA and chemotherapy	§5	10.3348/kjr.2016.17.4.497
**54**	Wu et al., 2017	Paclitaxel-loaded and A10-3.2 aptamer-targeted poly(lactide-co-glycolic acid) nanobubbles for ultrasound imaging and therapy of prostate cancer	Original study (preclinical, prostate)	Aptamer-targeted paclitaxel nanobubbles	§5	10.2147/ijn.s136032
**55**	Fan et al., 2016	Inhibition of prostate cancer growth using doxorubicin assisted by ultrasound-targeted nanobubble destruction	Original study (preclinical, prostate)	Doxorubicin delivery by targeted nanobubble destruction	§5	10.2147/ijn.s111808
**56**	Wang et al., 2015	Upregulation of ULK1 expression in PC-3 cells following tumor protein P53 transfection by sonoporation	Original study (in vitro, PC-3)	p53 transfection by sonoporation; ULK1 upregulation	§5	10.3892/ol.2015.3946
**57**	Wang et al., 2014	Preparation of Nanobubbles Carrying Androgen Receptor siRNA and Their Inhibitory Effects on Androgen-Independent Prostate Cancer when Combined with Ultrasonic Irradiation	Original study (preclinical, prostate)	Androgen-receptor siRNA nanobubbles with insonation	§5	10.1371/journal.pone.0096586
**58**	Escoffre et al., 2022	Delivery of anti-cancer drugs using microbubble-assisted ultrasound in digestive oncology: from preclinical to clinical studies	Review	USMB drug delivery in digestive oncology: preclinical to clinical	§5	10.1080/17425247.2022.2061459
**59**	El Kaffas et al., 2015	Biomechanical Effects of Microbubbles: From Radiosensitization to Cell Death	Review	Biomechanical microbubble effects from radiosensitization to cell death	§6	10.2217/fon.15.19
**60**	Al-Mahrouki et al., 2012	Bioeffects of Ultrasound-Stimulated Microbubbles on Endothelial Cells: Gene Expression Changes Associated with Radiation Enhancement In Vitro	Original study (in vitro)	Endothelial gene-expression changes underlying radiation enhancement	§6	10.1016/j.ultrasmedbio.2012.07.009
**61**	El Kaffas et al., 2018	Role of Acid Sphingomyelinase and Ceramide in Mechano-Acoustic Enhancement of Tumor Radiation Responses	Original study (preclinical, genetic)	ASMase/ceramide requirement for radioenhancement (ASMase-null)	§6; Table 1, 3; Table S3b	10.1093/jnci/djy011
**62**	Leong et al., 2023	Ultrasound-stimulated microbubbles enhanced vascular disruption in fractionated radiotherapy-treated tumours via ASMase activation	Original study (preclinical)	ASMase-mediated vascular disruption under fractionated radiotherapy	§6; Table 1	10.1242/dmm.049531
**63**	Al-Mahrouki et al., 2015	Ultrasound-stimulated microbubble enhancement of radiation treatments: endothelial cell function and mechanism	Original study (preclinical)	Endothelial function and mechanism of radiation enhancement	§6	10.18632/oncoscience.277
**64**	Al-Mahrouki et al., 2017	Microbubble-based enhancement of radiation effect: Role of cell membrane ceramide metabolism	Original study (preclinical)	Membrane ceramide metabolism in radiation enhancement	§6	10.1371/journal.pone.0181951
**65**	Sharma et al., 2023	Ultrasound-stimulated microbubbles enhancement of fractionated radiation for tumor treatment	Original study (in vivo, prostate)	USMB with fractionated radiotherapy, PC3 xenograft	§6, 9; Table 2, 3, 5; Table S3b	10.1186/s12885-023-10981-5
**66**	McNabb et al., 2020	Ultrasound-stimulated microbubble radiation enhancement of tumors: Single-dose and fractionated treatment evaluation	Original study (in vivo, prostate)	Single-dose and fractionated USMB radiation enhancement	§6, 9, 10; Table 2, 3; Table S3b	10.1371/journal.pone.0239456

No.	Source	Title	Source type	Role in the review	Cited in	DOI
**67**	Sharma et al., 2022	Focused Ultrasound Stimulation of Microbubbles in Combination With Radiotherapy for Acute Damage of Breast Cancer Xenograft Model	Original study (in vivo, breast)	FUS-stimulated microbubbles with radiotherapy, acute damage	§6; Table 3; Table S3b	10.1177/15330338221132925
**68**	Deng et al., 2018	Ultrasound-Stimulated Microbubbles Enhance Radiosensitization of Nasopharyngeal Carcinoma	Original study (preclinical, nasopharyngeal)	USMB radiosensitization in a non-prostate model	§6	10.1159/000492263
**69**	Fu et al., 2021	Ultrasound-stimulated microbubbles contributes to radiotherapy in esophageal carcinoma	Original study (preclinical, oesophageal)	USMB contribution to radiotherapy in a non-prostate model	§6	10.1139/bcb-2021-0061
**70**	McNabb et al., 2023	MR-guided ultrasound-stimulated microbubble therapy enhances radiation-induced tumor response	Original study (in vivo, image-guided)	MR-guided USMB radiation enhancement	§6; Table 3; Table S3b	10.1038/s41598-023-30286-8
**71**	Michon et al., 2022	Targeted Anti-Cancer Provascular Therapy Using Ultrasound, Microbubbles, and Nitrite to Increase Radiotherapy Efficacy	Original study (preclinical, vascular)	Provascular nitrite-carrying microbubbles with radiotherapy	§6; Table 1, 3; Table S3b	10.1021/acs.bioconjchem.1c00510
**72**	Milosevic et al., 2012	Tumor Hypoxia Predicts Biochemical Failure following Radiotherapy for Clinically Localized Prostate Cancer	Original clinical study	Tumour hypoxia predicts biochemical failure after prostate radiotherapy	§6 †	10.1158/1078-0432.ccr-11-2711
**73**	Bargh-Dawson et al., 2025	Radiotherapeutic enhancement using ultrasound-stimulated microbubbles: a critical review	Review (critical)	Critical appraisal of USMB radiotherapeutic enhancement	§6, 11; Table 1, 5	10.1080/09553002.2025.2498980
**74**	Sharma et al., 2023	Radiation combined with ultrasound and microbubbles: A potential novel strategy for cancer treatment	Review	Radiation combined with ultrasound and microbubbles	§6	10.1016/j.zemedi.2023.04.007
**75**	Widmark et al., 2019	Ultra-hypofractionated versus conventionally fractionated radiotherapy for prostate cancer: 5-year outcomes of the HYPO-RT-PC randomized, non-inferiority, phase 3 trial	Randomised clinical trial	Ultra-hypofractionated prostate radiotherapy (HYPO-RT-PC)	§6 †	10.1016/s0140-6736(19)31131-6
**76**	Rakotomalala et al., 2021	Hypoxia in Solid Tumors: How Low Oxygenation Impacts the Six Rs of Radiotherapy	Review	Hypoxia and the six Rs of radiotherapy	§6, 12	10.3389/fendo.2021.742215
**77**	Hu et al., 2024	Progress and preclinical application status of ultrasound microbubbles	Review	Progress and preclinical application status of USMB	§6	10.1016/j.jddst.2023.105312
**78**	Bai et al., 2013	Treatment of PC-3 cells with ultrasound combined with microbubbles induces distinct alterations in the expression of Bcl-2 and Bax	Original study (in vitro, PC-3)	USMB alone: Bcl-2/Bax alterations and apoptosis	§9; Table 1, 3; Table S3b	10.1007/s11434-013-5753-y
**79**	Chen et al., 2025	Modulating tumor interstitial fluid pressure using ultrasound and microbubble therapy: a preclinical study for enhanced drug delivery in cancer treatment	Original study (preclinical)	Modulation of tumor interstitial fluid pressure by USMB	§12; Table 1	10.1186/s12885-025-15218-1
**80**	Li et al., 2021	Tumor perfusion enhancement by ultrasound stimulated microbubbles potentiates PD-L1 blockade of MC38 colon cancer in mice	Original study (preclinical)	USMB perfusion enhancement potentiating PD-L1 blockade	§12; Table 1	10.1016/j.canlet.2020.10.046
**81**	Almasri et al., 2022	Synergistic enhancement of cell death by triple combination therapy of docetaxel, ultrasound and microbubbles, and radiotherapy on PC3 a prostate cancer cell line	Original study (in vitro, PC3)	Triple therapy: docetaxel + USMB + radiotherapy, Bliss synergy	§7, 8, 9; Table 2, 3; Table S3b	10.1016/j.heliyon.2022.e10213
**82**	Almasri et al., 2023	Radioenhancement with the Combination of Docetaxel and Ultrasound Microbubbles: In Vivo Prostate Cancer	Original study (in vivo, PC3)	Triple therapy in vivo with quantitative-ultrasound correlation	§7, 9, 10; Table 2, 3, 5; Table S3b	10.3390/pharmaceutics15051468

No.	Source	Title	Source type	Role in the review	Cited in	DOI
**83**	Mu et al., 2012	MR-guided pulsed high intensity focused ultrasound enhancement of docetaxel combined with radiotherapy for prostate cancer treatment	Original study (in vivo, LNCaP)	MR-guided pulsed FUS with docetaxel and radiotherapy	§7; Table 3; Table S3b	10.1088/0031-9155/57/2/535
**84**	Tu et al., 2024	Docetaxel radiosensitizes castration-resistant prostate cancer by downregulating CAV-1	Original study (preclinical)	Docetaxel radiosensitization of CRPC via CAV-1 downregulation	§7	10.1080/09553002.2023.2263553
**85**	Karshafian et al., 2009	Sonoporation by Ultrasound-Activated Microbubble Contrast Agents: Effect of Acoustic Exposure Parameters on Cell Membrane Permeability and Cell Viability	Original study (in vitro, mechanistic)	Acoustic exposure parameters governing permeability and viability	§8; Table 2, 5; Table S3b	10.1016/j.ultrasmedbio.2008.10.013
**86**	van Rooij et al., 2016	Viability of endothelial cells after ultrasound-mediated sonoporation: Influence of targeting, oscillation, and displacement of microbubbles	Original study (in vitro)	Endothelial viability after sonoporation: targeting and oscillation	§8	10.1016/j.jconrel.2016.07.037
**87**	Kim et al., 2013	Effects of Biophysical Parameters in Enhancing Radiation Responses of Prostate Tumors with Ultrasound-Stimulated Microbubbles	Original study (in vivo, prostate)	Biophysical parameters governing prostate tumor radiation response	Table 2; Table S3b	10.1016/j.ultrasmedbio.2013.01.012
**88**	Kotopoulis et al., 2022	SonoVue vs. Sonazoid vs. Optison: Which Bubble Is Best for Low-Intensity Sonoporation of Pancreatic Ductal Adenocarcinoma?	Original study (in vitro)	Comparison of SonoVue, Sonazoid, and Optison for sonoporation	§8, 11; Table 5	10.3390/pharmaceutics14010098
**89**	McCorkell et al., 2023	In Vitro Radioenhancement Using Ultrasound-Stimulated Microbubbles: A Comparison of Suspension and Adherent Cell States	Original study (in vitro)	Suspension versus adherent cell state in radioenhancement	§8	10.3390/radiation3030013
**90**	Klein et al., 2020	Effect of Treatment Sequencing on the Tumor Response to Combined Treatment With Ultrasound-Stimulated Microbubbles and Radiotherapy	Original study (in vivo, prostate)	Effect of USMB–radiotherapy treatment sequencing	§8; Table 2; Table S3b	10.1002/jum.15363
**91**	Wei et al., 2014	Combined treatment of PC-3 cells with ultrasound and microbubbles suppresses invasion and migration	Original study (in vitro, PC-3)	USMB suppression of invasion and migration (MMP-2/MMP-9)	§9; Table 3; Table S3b	10.3892/ol.2014.2310
**92**	Kim et al., 2014	Quantitative Ultrasound Characterization of Tumor Cell Death: Ultrasound-Stimulated Microbubbles for Radiation Enhancement	Original study (preclinical)	Quantitative-ultrasound characterization of tumor cell death	§10; Table 5	10.1371/journal.pone.0102343
**93**	Lee et al., 2012	Quantitative Ultrasound for the Monitoring of Novel Microbubble and Ultrasound Radiosensitization	Original study (preclinical)	Quantitative ultrasound for monitoring USMB radiosensitization	§10	10.1016/j.ultrasmedbio.2012.01.028
**94**	Sharma et al., 2021	In vivo assessment of prostate cancer response using quantitative ultrasound characterization of ultrasonic scattering properties	Original study (in vivo, prostate)	Quantitative-ultrasound assessment of prostate tumor response	§10	10.1186/s12885-021-08706-7
**95**	Zhou et al., 2022	Human clinical trial using diagnostic ultrasound and microbubbles to enhance neoadjuvant chemotherapy in HER2-negative breast cancer	Human clinical study	Diagnostic USMB with neoadjuvant chemotherapy, HER2-negative breast cancer	§10, 11; Table 4, 5	10.3389/fonc.2022.992774
**96**	Perera et al., 2020	Real time ultrasound molecular imaging of prostate cancer with PSMA-targeted nanobubbles	Original study (preclinical, prostate)	PSMA-targeted nanobubbles for molecular ultrasound imaging	§10	10.1016/j.nano.2020.102213
**97**	Uemura et al., 2013	Usefulness of perflubutane microbubble-enhanced ultrasound in imaging and detection of prostate cancer: phase II multicenter clinical trial	Human clinical trial (Phase II)	Perflubutane microbubble-enhanced ultrasound for prostate detection	§10	10.1007/s00345-012-0833-1

No.	Source	Title	Source type	Role in the review	Cited in	DOI
**98**	Bendjador et al., 2022	A theranostic 3D ultrasound imaging system for high resolution image-guided therapy	Original study (technical)	3D theranostic ultrasound system for image-guided therapy	§10; Table 5	10.7150/thno.71221
**99**	Meuillet et al., 2026	Development of Ultrasound Microbubbles and Phase Shift Microbubbles in Theranostics	Review	Development of microbubble and phase-shift agents in theranostics	§10	10.1002/wnan.70068
**100**	Dimcevski et al., 2016	A human clinical trial using ultrasound and microbubbles to enhance gemcitabine treatment of inoperable pancreatic cancer	Human clinical trial (Phase I)	USMB-enhanced gemcitabine, inoperable pancreatic cancer	§11; Table 4	10.1016/j.jconrel.2016.10.007
**101**	Kotopoulis et al., 2013	Treatment of human pancreatic cancer using combined ultrasound, microbubbles, and gemcitabine: A clinical case study	Human clinical case study	Combined ultrasound, microbubbles, and gemcitabine in pancreatic cancer	§11	10.1118/1.4808149
**102**	Moore-Palhares et al., 2024	Radiation enhancement using focussed ultrasound-stimulated microbubbles for breast cancer: A Phase 1 clinical trial	Human clinical trial (Phase 1)	FUS-stimulated microbubble radiation enhancement, breast cancer	§11; Table 4	10.1371/journal.pmed.1004408
**103**	Dasgupta et al., 2023	Novel MRI-guided focussed ultrasound stimulated microbubble radiation enhancement treatment for breast cancer	Human clinical study	MRI-guided FUS-stimulated microbubble radiation enhancement, breast	§11	10.1038/s41598-023-40551-5
**104**	Moore-Palhares et al., 2024	Radiation enhancement using focussed ultrasound-stimulated microbubbles for head and neck cancer: A phase 1 clinical trial	Human clinical trial (Phase 1)	FUS-stimulated microbubble radiation enhancement, head and neck	§11; Table 4	10.1016/j.radonc.2024.110380
**105**	Wessner et al., 2025	Microbubble-based Radiosensitization of Hepatocellular Carcinoma: Evaluation of Safety and Efficacy in a Phase II Randomized Trial	Human randomized trial (Phase II)	Microbubble-based radiosensitization of hepatocellular carcinoma	§11; Table 4	10.1148/radiol.250083
**106**	Haram et al., 2025	Treatment of inoperable pancreatic adenocarcinoma with focused ultrasound and microbubbles in patients receiving chemotherapy	Human clinical study (randomized pilot)	FUS and microbubbles with chemotherapy, pancreatic adenocarcinoma	Table 4	10.1016/j.wfumbo.2025.100080
**107**	Keller et al., 2021	Safety of Image-Guided Treatment of the Liver with Ultrasound and Microbubbles in an in Vivo Porcine Model	Original study (in vivo, porcine)	Safety of image-guided liver treatment with ultrasound and microbubbles	§11; Table 5	10.1016/j.ultrasmedbio.2021.07.003
**108**	Eckardt et al., 2026	From development to regulation: Translational pipeline results for molecularly targeted polymeric microbubbles	Original study (translational)	Development-to-regulation pipeline for targeted polymeric microbubbles	§11; Table 5	10.1016/j.nantod.2026.103149
**109**	Yang et al., 2025	A Review on the Structure-Response-Efficacy Optimization of Ultrasound-Responsive Micro/Nanobubbles for Cancer Therapy	Review	Structure–response–efficacy optimisation of responsive micro/nanobubbles	§11	10.3390/pharmaceutics17111378
**110**	Lin et al., 2026	Beyond total release: Fragment-resolved metrics for ultrasound microbubble-mediated drug delivery	Original study (methodological)	Fragment-resolved metrics for USMB drug release	§11	10.1016/j.biopha.2026.119243
**111**	Sarkar et al., 2026	Versatile potential of ultrasound mediated microbubble for biomedical applications: Current trends and emerging strategies	Review	Biomedical applications of ultrasound-mediated microbubbles	§11	10.1016/j.jddst.2026.108199
**112**	Dietrich et al., 2020	Guidelines and Good Clinical Practice Recommendations for Contrast Enhanced Ultrasound (CEUS) in the Liver-Update 2020-WFUMB in Cooperation with EFSUMB, AFSUMB, AIUM, and FLAUS	Guideline	WFUMB/EFSUMB good clinical practice recommendations for CEUS	§11 †	10.1055/a-1177-0530
**113**	Almushayt, 2026	Barriers to the Broader Use of Contrast-Enhanced Ultrasound in Saudi Healthcare and Medical Research	Review / commentary	Barriers to broader clinical adoption of contrast-enhanced ultrasound	§11; Table 5	10.1055/a-2899-0967
**114**	de Maar et al., 2021	Ultrasound-Mediated Drug Delivery With a Clinical Ultrasound System: In Vitro Evaluation	Original study (in vitro)	Ultrasound-mediated delivery using a clinical ultrasound system	§11; Table 5	10.3389/fphar.2021.768436
**115**	Xu et al., 2019	Translational Prospects of ultrasound-mediated tumor immunotherapy: Preclinical advances and safety considerations	Review	Translational prospects and safety of ultrasound-mediated immunotherapy	§12	10.1016/j.canlet.2019.06.017

No.	Source	Title	Source type	Role in the review	Cited in	DOI
**116**	Chen et al., 2026	Recent advances in ultrasound-targeted nanobubbles combined with cancer immunotherapy: Mechanisms, applications, and challenges	Review	Ultrasound-targeted nanobubbles combined with immunotherapy	§12	10.1016/j.fmre.2024.10.017
**117**	Rivera et al., 2024	A Review of Ultrasound-Mediated Checkpoint Inhibitor Immunotherapy	Review	Ultrasound-mediated checkpoint-inhibitor immunotherapy	§12	10.1016/j.ultrasmedbio.2023.08.019
**118**	Liu et al., 2025	Ultrasound-responsive micro/nanobubbles based intelligent theranostic systems for precision tumor therapy	Review	Responsive micro/nanobubble theranostic systems	§12	10.1186/s12951-025-03959-x
**119**	Perera et al., 2024	Ultrasound-mediated drug-free theranostics for treatment of prostate cancer	Original study (preclinical, prostate)	Ultrasound-mediated drug-free theranostics in prostate cancer	§12	10.1016/j.bioactmat.2023.12.012
**120**	Li, 2026	Nanotechnology in Prostate Cancer: PSMA-Targeted Nanoplatforms, TME-Responsive Therapy, Immunomodulation, and Clinical Translation Challenges	Review	PSMA-targeted nanoplatforms and translation challenges in prostate cancer	§12	10.2147/ijn.s629751
**121**	Kerkmeijer et al., 2021	Focal Boost to the Intraprostatic Tumor in External Beam Radiotherapy for Patients With Localized Prostate Cancer: Results From the FLAME Randomized Phase III Trial	Randomised clinical trial	Focal boost to the intraprostatic lesion (FLAME trial)	§12 †	10.1200/jco.20.02873

† Cited for contextual discussion only and not charted as therapeutic evidence; see Section 2.3 of the manuscript and Supplementary Table S4. These seven references, together with the 111 charted sources, gave the 118 references in the reference list as originally submitted. ‡ Contextual reference added during the peer-review revision at reviewer suggestion; identified outside the systematic search and not charted as therapeutic evidence. With these three additions [28–30], the final reference list contains 121 references.

Table S3b. Per-source charted-data extraction table (experimental evidence base). Charted study-level data for the experimental and mechanistic sources that form the primary evidence base of the review (the sources realised in manuscript Tables 2 and 3). Human clinical studies are charted separately in manuscript Table 4. Review, mechanistic-background, clinical-context, and methodological references form the contextual reference base and are cited in the main reference list rather than charted with study-level parameters. NR = not reported / not extractable from the source; - = not applicable to that study.

Source	DOI	Evidence tier	Model / cell line	Intervention (vs comparators)	US frequency / pressure / pulse	Microbubble	Chemo (dose)	Radiation (dose/fx)	Principal charted outcome
Almasri et al., 2022	10.1016/j.heliyon.2022.e10213	In vitro (prostate)	PC3, suspension	USMB+DTX+XRT vs single/double	500 kHz / 580 kPa PNP / 10 µs, 60 s	Definity, 2% v/v	DTX, 0.001–0.1 nM	2 Gy	Triple → ~2% clonogenic viability; Bliss synergy, parameter-dependent
Almasri et al., 2023	10.3390/pharmaceutics15051468	In vivo mouse (prostate)	PC-3 xenograft, SCID	USMB+DTX+XRT vs single/double	500 kHz / 350 then 580 kPa PNP / 16-cycle burst (32 µs), 3 kHz PRF, 2 min	Definity, 2 µL/g	DTX, 5 mg/kg	8 Gy single	Cell death 83% / apoptosis 71% vs 16% / 14% (XRT); QUS correlated
Sharma et al., 2023	10.1186/s12885-023-10981-5	In vivo mouse (prostate)	PC3 xenograft	Fractionated XRT ± USMB	500 kHz / 570 kPa PNP / 50 ms bursts (3 kHz PRF) every 2 s, 5 min	Definity, 3% v/v	-	40 Gy / 20 fx	Tumour shrinkage + vascular disruption only with combination
McNabb et al., 2020	10.1371/journal.pone.0239456	In vivo large-animal (prostate)	PC3 xenograft, rabbits	USMB + XRT	NR / NR / NR	NR	-	8 Gy single; or 2 Gy ×5/wk × 3 wk	Vascular shutdown, growth delay; 87% vs 14% survival at 21 d
Klein et al., 2020	10.1002/jum.15363	In vivo (prostate)	Prostate xenograft	USMB–XRT sequencing (0–24 h)	NR / NR / NR	Lipid microspheres	-	8 Gy	Largest effect ~6 h before XRT (sequencing differences NS)
Goertz et al., 2012	10.1371/journal.pone.0052307	In vivo mouse (prostate)	PC3 xenograft	Antivascular USMB + DTX	1 MHz / 1.65 MPa / 50 ms bursts (0.00024 duty cycle), 5 min	NR	DTX, 5 mg/kg weekly ×4	-	Acute perfusion loss, ↑necrosis/apoptosis 24 h; prolonged survival
Mu et al., 2012	10.1088/0031-9155/57/2/535	In vivo mouse (prostate)	LNCaP orthotopic	MR-guided pulsed FUS + DTX + XRT	Pulsed FUS (MR-guided) / NR / NR	NR	DTX, 5 mg/kg	4 Gy or 6 Gy single fx	Greatest growth inhibition with triple; small groups,

Source	DOI	Evidence tier	Model / cell line	Intervention (vs comparators)	US frequency / pressure / pulse	Microbubble	Chemo (dose)	Radiation (dose/fx)	Principal charted outcome
									inconsistent combination effect
Karshafian et al., 2010	10.1109/ULTSYM.2010.5935914	In vitro (prostate)	PC3 / MDA-MB-231, suspension	USMB + DTX (varied pressure/order)	500 kHz / 240 & 580 kPa / 30 s, 3 kHz PRF	DA04 (Artenga), 0.5% v/v	DTX (Taxotere; dose NR)	-	Enhanced kill; smaller effect in chemoresistant PC3
Karshafian et al., 2009	10.1016/j.ultrasmedbio.2008.10.013	In vitro (mechanistic)	KHT-C	Parameter optimisation (tracer uptake/viability)	500 kHz / 570 kPa (0.05–3.5 MPa tested) / 8 µs, 3 kHz PRF, 12 s	Definity, 3.3% v/v	- (model tracer)	-	Permeability↑ / viability↓ with pressure, PRF, pulse, time; therapeutic-ratio optimum
Kim et al., 2013	10.1016/j.ultrasmedbio.2013.01.012	In vivo mouse (prostate)	PC-3 xenograft	USMB + XRT (dose–response)	500 kHz / 250 / 570 / 750 kPa / NR	Definity, 8 / 80 / 1000 µL/kg	-	0 / 2 / 8 Gy	Radioenhancement scales with pressure, MB concentration, and dose
Luo et al., 2022	10.1080/10717544.2022.2115163	Comparator (solid tumor)	VX2 rabbit	Sonoporation/USMB + gemcitabine (timing)	3 MHz / 0.26 MPa / 5-cycle bursts, 0.125% duty	Lipid MB	Gemcitabine (dose NR)	-	Highest intratumoral drug when USMB immediately follows chemotherapy (2.83× control)
El Kaffas et al., 2018	10.1093/jnci/djy011	Mechanistic (genetic)	MCA/129 fibrosarcoma, ASMase⁻/⁻ vs WT	USMB + fractionated XRT	NR / NR / NR	NR	-	Fractionated XRT (dose NR)	Radioenhancement and vascular disruption abolished in ASMase⁻/⁻ (ceramide proof)
Michon et al., 2022	10.1021/acs.bioconjchem.1c00510	Mechanistic (vascular)	Tumour, mice	USMB + nitrite (provascular) + XRT	NR / NR / NR	Nitrite-carrying MB	-	XRT (dose NR)	Provascular reoxygenation enhances radiation (opposite direction to antivascular shutdown)
Sharma et al., 2022	10.1177/15330338221132925	Comparator (solid tumor)	MDA-MB-231 breast xenograft	FUS + MB + XRT	NR / NR / NR	NR	-	8 Gy	↑Cell death, ↓vascularity (CD31/Doppler), ↑ceramide, ↑hypoxia
Kotopoulis et al., 2014	10.1007/s11307-013-0672-5	Comparator (solid tumor)	MIA PaCa-2 orthotopic pancreatic	Sonoporation/USMB + gemcitabine	NR / NR / NR	NR	Gemcitabine (dose NR)	-	Reduced primary tumor burden
McNabb et al., 2023	10.1038/s41598-023-30286-8	In vivo mouse (image-guided)	Tumour xenograft	MR-guided USMB + XRT	MR-guided / NR / NR	NR	-	XRT (dose NR)	Independent replication of USMB radioenhancement under MR guidance
Bai et al., 2013	10.1007/s11434-013-5753-y	In vitro (prostate)	PC-3	USMB alone	NR / NR / NR	NR	-	-	Apoptosis via ↓Bcl-2 / ↑Bax
Wei et al., 2014	10.3892/ol.2014.2310	In vitro (prostate)	PC-3	USMB alone	NR / NR / NR	NR	-	-	Suppressed invasion/migration via ↓MMP-2 / MMP-9

Charting framework (applied to all sources). The variables charted for each source were: source identifier (first author, year, DOI); evidence tier; model system and host; cell line; intervention and comparator; ultrasound frequency, acoustic pressure, and pulse/insonation characteristics; microbubble agent and concentration; chemotherapy agent and dose; radiation dose and fractionation; treatment sequencing; reported outcomes (clonogenic survival/viability, apoptosis/necrosis, vascular endpoints, tumor growth/survival, imaging biomarkers); synergy-assessment method and result where reported; and bibliographic-verification status (publisher/PubMed/Crossref).

Table S4. Records Screened and Per-Stage Charting Counts

Funnel of the charted-evidence set corresponding to Figure 1. All record counts below derive from the structured query executed in Scopus, together with the three supplementary sources identified through reference-list and citation searching; the verification resources listed in Table S2.1 contributed no records to this funnel. Counts are internally consistent: 945 − 606 = 339; 339 − 231 = 108; 108 + 3 = 111. The high duplicate count reflects export from multiple overlapping search blocks (prostate-specific, mechanistic, and translational) before de-duplication, so that sources retrieved by more than one block appeared repeatedly in the combined export. Following peer review, three further contextual references [28–30] were added, bringing the final reference list to 121 references; they are marked ‡ in Table S3a and do not alter the screening funnel.

Stage	Count (n)	Notes
Records retrieved and extracted	945	Combined export of the primary and supplementary blocks (Table S2); title, abstract, and structured methods/results/conclusions recorded for each.
Duplicate records removed	606	Removed during de-duplication of the combined export.
Unique records screened against eligibility criteria	339	Screened against the pre-defined criteria in Section 2.2.
Records not meeting eligibility criteria or retained only for background context	231	Predominantly imaging-only, formulation-/manufacturing-only, or non-oncological reports; some retained as background but not treated as therapeutic evidence.
Sources meeting eligibility criteria	108	Charted as primary evidence (Tables 1–5) or included in the contextual reference base and cited.
Additional sources identified through supplementary searching	3	Identified through supplementary citation/database searching after the main screen.
**Sources included in charted synthesis and contextual reference base**	**111**	Comprises original studies together with review, mechanistic, and clinical-context references.
Further contextual references cited outside the charted set	7	Reporting-guidance and risk-of-bias instruments, prostate radiotherapy guideline and focal-boost context, international contrast-enhanced-ultrasound guidance, and hypoxia and fractionation context cited in the discussion and future-directions sections; not charted as therapeutic evidence.
Contextual references added during peer-review revision	3	Reviewer-suggested references [28–30]; identified outside the systematic search; not charted as therapeutic evidence.
**Total references in the final reference list**	121	111 charted/contextual sources + 7 further contextual references + 3 contextual references added during peer-review revision [28–30].

Table S5. Proposed Minimum Reporting Checklist for Acoustic, Microbubble, Drug, and Radiation Parameters

Reporting of the items below, in the spirit of established reporting instruments, would make future preclinical and clinical USMB chemo- and radioenhancement studies comparable in the respects that currently frustrate cross-study synthesis (Section 8 of the manuscript). The checklist is proposed as a starting point for consensus rather than as a finished standard; "*in situ*" pressure denotes the pressure at the target after accounting for attenuation, with the derating method stated.

Item	What should be reported
Transducer and geometry	Transducer type, aperture, focal geometry, and coupling path.
Center frequency	Operating frequency (MHz).
Acoustic pressure	Peak-negative pressure *in situ* (MPa), with calibration method and derating assumptions stated.
Calibration and derating	Hydrophone or equivalent calibration of the acoustic field, the derating method and tissue attenuation coefficient applied, and whether reported pressures are free-field or in situ values.
Mechanical index	Mechanical index at treatment settings.
Pulse scheme	Pulse duration or cycle count, burst length, and waveform.
Pulse-repetition frequency	PRF (Hz or kHz).
Duty cycle	Explicit duty cycle (%).
Exposure duration	Total insonation time and any interleaving or scanning scheme.
Cavitation monitoring	Whether cavitation activity was verified (e.g., passive cavitation detection) and how.
Microbubble agent	Agent, shell and gas composition where known, size distribution, formulation, and preparation/reconstitution procedure.

Microbubble dose	Concentration or dose, diluent, administration route and rate, and whether given as a bolus or an infusion.
Microbubble timing	Timing of microbubble administration relative to insonation.
Drug parameters	Agent, dose, route, schedule, and timing relative to insonation and to radiotherapy.
Radiation parameters	Dose, fractionation, beam quality, and timing relative to insonation and to chemotherapy.
Model state	Cell line and cell state (suspension vs. adherent) *in vitro*; tumor model, site, and size *in vivo*; immune status of the host (immunodeficient vs. immunocompetent); anesthesia.
Target coverage	Focal dimensions and treated volume, beam–target geometry, and whether whole-target coverage was achieved or estimated.
Outcome assays	Endpoints, assay timepoints, and quantification methods, including the timing of each endpoint relative to treatment.
Thermal monitoring	Whether tissue heating was measured or modelled, and by what method, where thermal effects are plausible.
Toxicity monitoring	Prospective toxicity endpoints collected, their grading scale, and the monitoring schedule.
Normal-tissue assessment	Assessment of normal tissues within the acoustic field, including any histological or functional evaluation of organs adjacent to the target.
